# Calmodulin acts as a selective switch in FGF12A-mediated regulation of the cardiac sodium channel Nav1.5

**DOI:** 10.1016/j.jbc.2026.113565

**Published:** 2026-09-17

**Authors:** Lucy Woodbury, Paweorn Angsutararux, Martina Marras, Emily Wagner, Carlota Abella, Anna Li, Jonathan R. Silva

**Affiliations:** 1 Department of Biomedical Engineering, McKelvey School of Engineering, Washington University in St Louis, St Louis, Missouri, USA; 2 Department of Pharmacology, School of Medicine, UC Davis, Davis, California, USA

**Keywords:** auxiliary subunits, calmodulin, cardiac action potential, electrophysiology, FGF12A, Nav1.5 channel

## Abstract

The voltage-gated Na^+^ channel (Nav1.5) is responsible for the rapid depolarization of the cardiac action potential and is associated with the initiation of neuronal action potentials in the central nervous system. Nav1.5 channel gating is modulated by its C-terminal domain and the multiple auxiliary proteins that are bound to it, including calmodulin (CaM) and intracellular fibroblast growth factors (iFGFs). A non-canonical CaM-binding site was recently discovered on the N-terminus of iFGF A-splice variants. To test whether FGF12A, CaM-bound or not, regulates Nav1.5 gating, we performed cut-open Vaseline gap voltage-clamp on Nav1.5 heterologously expressed in *Xenopus laevis* oocytes. We observed that only CaM-bound FGF12A (WT FGF12A) shifted the voltage-dependence of steady-state inactivation of Nav1.5. The effect of CaM-bound FGF12A was amplified when CaM was absent from the Nav1.5 C-terminal domain (IQ/AA variant) or when the channel carried Long QT syndrome Type 3 variants. CaM-bound FGF12A additionally shifted the activation of voltage-sensing domain IV within the Nav1.5 channel as assessed by voltage-clamp fluorometry. Conversely, the ability of FGF12A to reduce late sodium current, I_Na,L_, did not depend on CaM binding. We conclude that FGF12A regulates Nav1.5 through two distinct mechanisms: CaM-bound FGF12A is required to shift the voltage-dependence of inactivation, but CaM binding is not necessary for the inhibition of pathogenic I_Na,L_. These findings reveal that CaM acts as a selective switch within the FGF12A regulatory complex, enabling voltage-dependent inactivation shifts while late current suppression proceeds independently. This bifurcated architecture is likely conserved across A-type iFGF interactions with Nav channel alpha-subunits.

Voltage-gated Na^+^ (Nav) channels are responsible for the initiation of the action potential in excitable cells including neurons and myocytes. The Nav1.5 sodium channel, encoded by the *SCN5A* gene, is the most prevalent Nav channel in cardiomyocytes and is responsible for the rapid post-stimulus depolarization of the myocyte (1). The Nav1.5 channel also plays a vital role in the initiation of neuronal action potentials in the central nervous system (2, 3). Nav1.5 contains four homologous repeats (I-IV) each comprising six transmembrane helices (S1-S6) with the first four defining the voltage-sensing domain (VSD). The S4 helix carries multiple positive charges, which translocate in response to depolarization of the cell membrane to open and close the channel (1, 4, 5, 6). Nav1.5 gating has been described in terms of closed (deactivated), open (activated), and inactivated states (7, 8) where channels transition from closed to open in response to depolarizing changes in membrane potential and to inactivated when an elevated membrane potential is prolonged. From the closed state, strong depolarization of the plasma membrane will activate the first three VSDs (I-III). Notably, VSD-IV is not required for channel-pore opening, but it is required for inactivation (9, 10). VSD-IV activation exposes a hydrophobic pocket near the intracellular pore entrance, allowing the isoleucine-phenylalanine-methionine (IFM) motif on the DIII-DIV linker to bind (5, 11). IFM binding drives closure of the S5-S6 helices, inactivating the channel and allowing membrane repolarization (12). The C-terminal domain (CTD) of Nav1.5 has been implicated in modulating both activation and inactivation kinetics (13). Recent structures of the Nav1.5 channel in its open state have shown the channel CTD interacting with the DIII-DIV linker, supporting a C-terminal role in inactivation regulation (14, 15, 16, 17).

Numerous variants within *SCN5A* are known to predispose patients to cardiac arrhythmia. This pathology can arise when variants alter the peak and late components of the Na^+^ current (I_Na_) causing either a gain or loss of function. Long QT syndrome Type 3 (LQT3) arises from the prolongation of ventricular repolarization that manifests as a prolonged ventricular myocyte action potential and QT interval of an ECG and is linked to gain-of-function mutations that typically affect the late Na^+^ current (I_Na,L_). Normally, I_Na,L_ is relatively small compared to the peak I_Na_ (0.1–1%) and variants that significantly increase I_Na,L_ bring excess positive charges into the myocyte that prolong the action potential, leading to proarrhythmic triggered activity that includes early and delayed afterdepolarizations (18). Beyond cardiac pathology, SCN5A variants have been linked to neurological phenotypes as well. Loss-of-function mutations within *SCN5A* are usually associated with Brugada syndrome, which has over 300 associated variants (19) that can cause arrhythmia *via* slowed conduction and dispersion of repolarization. *SCN5A* variants that cause Brugada syndrome have also been reported to cause epilepsy (20, 21, 22). In addition, patients with LQT3 variants (such as G1631D and P1332L) often have been reported to suffer from seizures (23). The connection between *SCN5A* variants and epilepsy suggests an important role in neuronal electrophysiology for the channel. Immunostaining for Nav1.5 in mouse brain slices has additionally shown expression in the cerebral cortex and hypothalamus (24, 25).

Multiple subunits interact with Nav1.5 to regulate its function and pharmacology (26). The β1 (*SCN1B*) subunit alters the voltage dependence of VSD-III and VSD-IV activation, Nav1.5 gating kinetics, and anti-arrhythmic drug affinity (27, 28, 29, 30, 31, 32). Calmodulin (CaM) alters membrane trafficking and modulates the voltage sensing kinetics of multiple channels including KCNQ1, Nav1.5, and Cav1.2 (33, 34, 35, 36, 37, 38). The CaM protein itself is a ubiquitous Ca^2+^ sensor made up of four EF-hands arranged in a dumbbell-like manner. CaM conformation and binding orientation are altered when Ca^2+^ is bound on its C and N-lobes. For certain targets, such as Cav2.1 and CaM-dependent protein kinase II, CaM must be saturated with Ca^2+^ at all four EF-hands to bind (39). In contrast, apo-CaM can interact with the Nav1.5 CTD *via* the IQ motif at residues 1908-09 (38, 39, 40). CaM binding to Nav1.5 has been proposed to be protective against increased I_Na,L_ and allows for increased peak channel opening (41, 42).

Intracellular fibroblast growth factors (iFGFs), also known as fibroblast homologous factors, are a family of proteins with distinct splice isoforms (*e.g.*, FGF12A and FGF12B). Unlike canonical FGFs, iFGFs lack the N-terminal secretion sequence and do not bind FGF receptors but do modulate voltage-gated Na^+^ and Ca^2+^ channels (43). iFGFs interact with the Nav1.5 channel through the iFGF β-trefoil at the EF-hand of the Nav1.5 CTD (44, 45). The interaction between FGF12 and Nav1.5 is known to induce a depolarizing shift in the steady-state inactivation (SSI) of the channel, leading to a reduction in the number of channels inactivated at a resting membrane potential (45, 46, 47). FGF12A has also been shown to reduce pathogenic I_Na,L_ carried by Nav1.5 channels (48) suggesting an anti-arrhythmic benefit of FGF12A (49). FGF12A and Nav1.5 have also been detected in murine neurons, specifically within both excitatory and inhibitory cells in the hippocampus (24, 50, 51, 52), indicating that this interaction is physiologically relevant for both cardiac and neuronal action potentials.

All A-splice variants of iFGFs have recently been shown to have a noncanonical CaM binding site on the N-terminal portion of the protein (53). The CaM binding region on FGF12A has a K_d_ of 107 nM, raising the possibility that multiple CaMs can simultaneously occupy the Nav1.5 CTD complex (53). Whether FGF12A-bound CaM independently regulates Nav1.5 gating kinetics, and which specific properties it controls, has not been established. Here we test whether CaM bound to the FGF12A N-terminus is required for FGF12A-mediated control of Nav1.5 gating (48).

## Results

### A-splice variants of iFGFs have a noncanonical CaM binding region which affects Nav1.5 function

To test whether CaM bound to FGF12A is required for FGF12A-mediated control of Nav1.5 gating, we performed cut-open voltage clamp recordings. FGF12A del CaM lacks the entire CaM binding region (residues 38–66) on the N-terminus, while FGF12A scram CaM retains a similar structure but removes the key CaM contact residues (Fig. 1*B*). We conducted cut-open voltage clamp recordings with Nav1.5 expressed alone or with: WT FGF12A (*red*), FGF12A del CaM (*blue*), and FGF12A scram CaM (*orange*) (Fig. 1*C*). Under these recording conditions, WT FGF12A is expected to be CaM-bound while FGF12A del CaM and FGF12A scram CaM have reduced/negligible CaM binding (see Limitations section).

**Figure 1:**
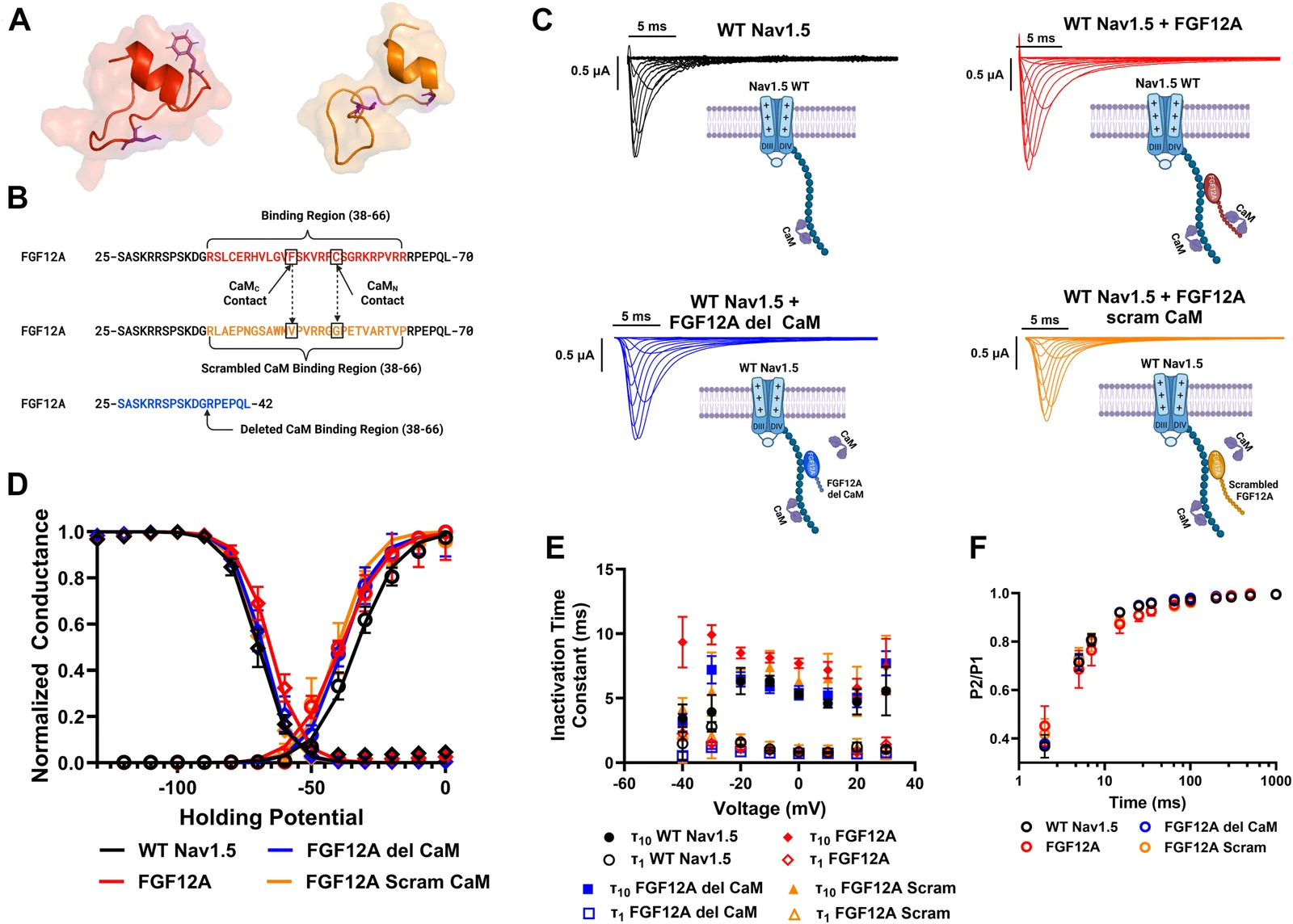
**CaM binds to FGF12A non-canonically to confer shifts in inactivation kinetics.***A*, predictive peptide structure of the CaM binding region of WT FGF12A (*red*) and the scrambled CaM binding region (*orange*). Both have analogous secondary structures; however, the proposed CaM contact points (*highlighted in purple*) are altered. *B*, sequence of CaM binding region described in Mahling *et al.* 2021. *Red*: WT FGF12A, *Blue*: deleted CaM binding region (FGF12A del CaM), *Orange*: scrambled CaM binding region (FGF12A Scram CaM). The corresponding CaM contact points are highlighted. *C*, representative traces with corresponding auxiliary subunit schematics. *D*, mean normalized ±SEM conductance-voltage (GV) and SSI curves for WT Nav1.5 and the addition of: FGF12A, FGF12A del CaM, and FGF12A Scram CaM. Curves are fitted single Boltzmann functions. The absence of CaM on FGF12A shows a slight shift in the inactivation of the Nav1.5 (Table 1). Comparisons were made with an ordinary one-way ANOVA with Tukey's multiple comparison test. *E*, mean ± SEM τ_1_ and τ_10_ inactivation time constants of I_Na_ decay fitted to a double exponential described in Experimental procedures. WT FGF12A showed a marked increase in the slow time component at multiple holding potentials. *F*, mean ± SEM recovery from inactivation from protocol described in Methods. CaM, calmodulin; SSI, steady-state inactivation.

Co-injection of WT FGF12A resulted in a small depolarizing shift in SSI (+5.39 mV ± 1.55, *p* = 0.0462) and a hyperpolarizing shift in GV curve (−8.23 mV ± 2.07, *p* = 0.0005) (Fig. 1*D* and Table 1), consistent with previous observations (46). Inactivation was well-fit with two exponentials with time constants on the order of 10 ms and 1 ms. The 10 ms inactivation time constant (τ_10_) was significantly increased with WT FGF12A co-injection (+5.99 ms ± 1.40, *p* = 0.0052, Fig. 1*E*). Conversely, removing the CaM binding region from FGF12A did not change SSI V_1/2_ (−0.500 mV ± 0.992, *p* = 0.618) (Table 1). No changes were observed in recovery from inactivation with the addition of either WT FGF12A or FGF12A del CaM (Fig. 1*F*).

**Table 1:** Properties of I_Na_ variants with the addition of WT FGF12A or FGF12A del CaM

Variant	GV activation (n)	V_1/2, act_ (mV)	±SEM	SSI inactivation (n)	V_1/2, inact_ (mV)	±SEM	I_Na,L_ (n)	% peak current (−30 mV)	±SEM
Nav1.5	17	−31.7	1.35	18	−67.5	0.473	5	0.987	0.242
Nav1.5 + FGF12A	11	−39.93	1.49	13	−62.1	0.79	5	1.51	0.010
Nav1.5 + FGF12A del CaM	11	−40.4	3.13	14	−68	0.95	10	1.71	0.238
Nav1.5 + FGF12A Scram CaM	6	−40.3	3.60	6	−69.2	0.863	7	0.459	0.092
IQ/AA	12	−20.3	1.58	10	−78.1	0.827	5	4.69	0.354
IQ/AA + FGF12A	11	−35.4	2.87	13	−65.9	1.23	5	2.36	0.498
IQ/AA + FGF12A del CaM	17	−29.3	1.05	5	−79	3.15	4	1.94	0.620
IQ/AA + FGF12A + CaM Q136P	6	−33	0.817	6	−58.5	1.12	6	1.62	0.314
IQ/AA + FGF12A del CaM + CaM Q136P	7	−29.6	2.41	4	−78.5	1.94	5	1.56	0.144
IQM	4	−29.3	4.52	4	−40	4.70	5	13.6	2.12
IQM + FGF12A	5	−39.4	1.61	5	−40	1.20	5	6.74	0.912
IQM + FGF12A del CaM	8	−28.9	2.38	8	−37.3	2.63	5	7.43	0.859
K1493E	4	−27.3	3.43	5	−81.8	0.970	5	1.09	0.506
K1493E + FGF12A	7	−29.9	1.44	7	−83.4	1.99	7	0.811	0.257
K1493E + FGF12A del CaM	5	−37.2	1.99	5	−84.6	0.872	7	1.36	0.356
S1904L	10	−20.1	2.51	10	−71.9	1.61	10	2.32	0.218
S1904L + FGF12A	10	−20.6	1.89	10	−58.7	1.13	7	1.18	0.175
S1904L + FGF12A del CaM	10	−18	3.31	10	−69.5	0.764	7	0.674	0.257
Y1795C	6	−21.8	3.4	6	−77.2	1.14	6	3.42	0.550
Y1795C + FGF12A	8	−35.6	1.92	7	−70.9	0.705	5	0.988	0.171
Y1795C + FGF12A del CaM	9	−23.8	2.59	9	−79.31	0.637	8	0.993	0.145

CaM, calmodulin.

Values are either means with ±SEM or the number (n) of cells analyzed in each group. Percent peak current was measured at −30 mV (see Experimental procedures).

Because the deleted region in FGF12A del CaM (residues 38–66) represents a significant portion of the N-terminus, we tested whether FGF12A scram CaM, which maintains similar length and predicted structure but removes the CaM contact residues, produces equivalent effects. A predictive peptide model of residues 38 to 66 of WT FGF12A revealed a short alpha-helix section followed by a disordered protein chain, and the scrambled FGF12A showed an analogous structural pattern, as assessed by PEP-FOLD3 modeling (Fig. 1*A*). No significant changes in FGF12A scram CaM’s effects compared to the FGF12A del CaM protein were detected for both the GV (+0.13 mV ± 4.08, *p* = 0.808) and SSI V_1/2_ (−1.22 mV ± 1.98, *p* = 0.418) (Fig. 1, *D*–*F* and Table 1). We therefore conclude that the loss of SSI shift and slowed inactivation in FGF12A del CaM reflects absent CaM binding, not a structural consequence of the deletion itself. Given their equivalence in this experiment, the following experiments used FGF12A del CaM to evaluate the effects of CaM removal from FGF12A.

### Removing CaM from the Nav1.5 CTD unmasks the full CaM-dependent effect of FGF12A on Nav1.5 gating

Results from Figure 1 show that FGF12A can only shift WT Nav1.5 inactivation when CaM is bound to the FGF12A N-terminus. Therefore, we considered whether the removal of CaM from the Nav1.5 CTD would amplify the FGF12A effect, given that the only CaM present in the Nav1.5 complex would be associated with WT FGF12A. We proceeded to test this hypothesis by using the IQ(1908-09)/AA variant that inhibits CaM binding to Nav1.5 (54) (Fig. 2*A*). The IQ/AA variant of Nav1.5 independently has large hyperpolarizing and depolarizing shifts in its SSI (−10.6 mV ± 1.68, *p* < 0.0001) and GV V_1/2_ (+11.4 mV ± 2.65, *p* = 0.003) respectively and significantly increases the I_Na,L_ (+3.7% ± 0.562, *p* < 0.0001) compared to WT Nav1.5 (Table 1).

**Figure 2:**
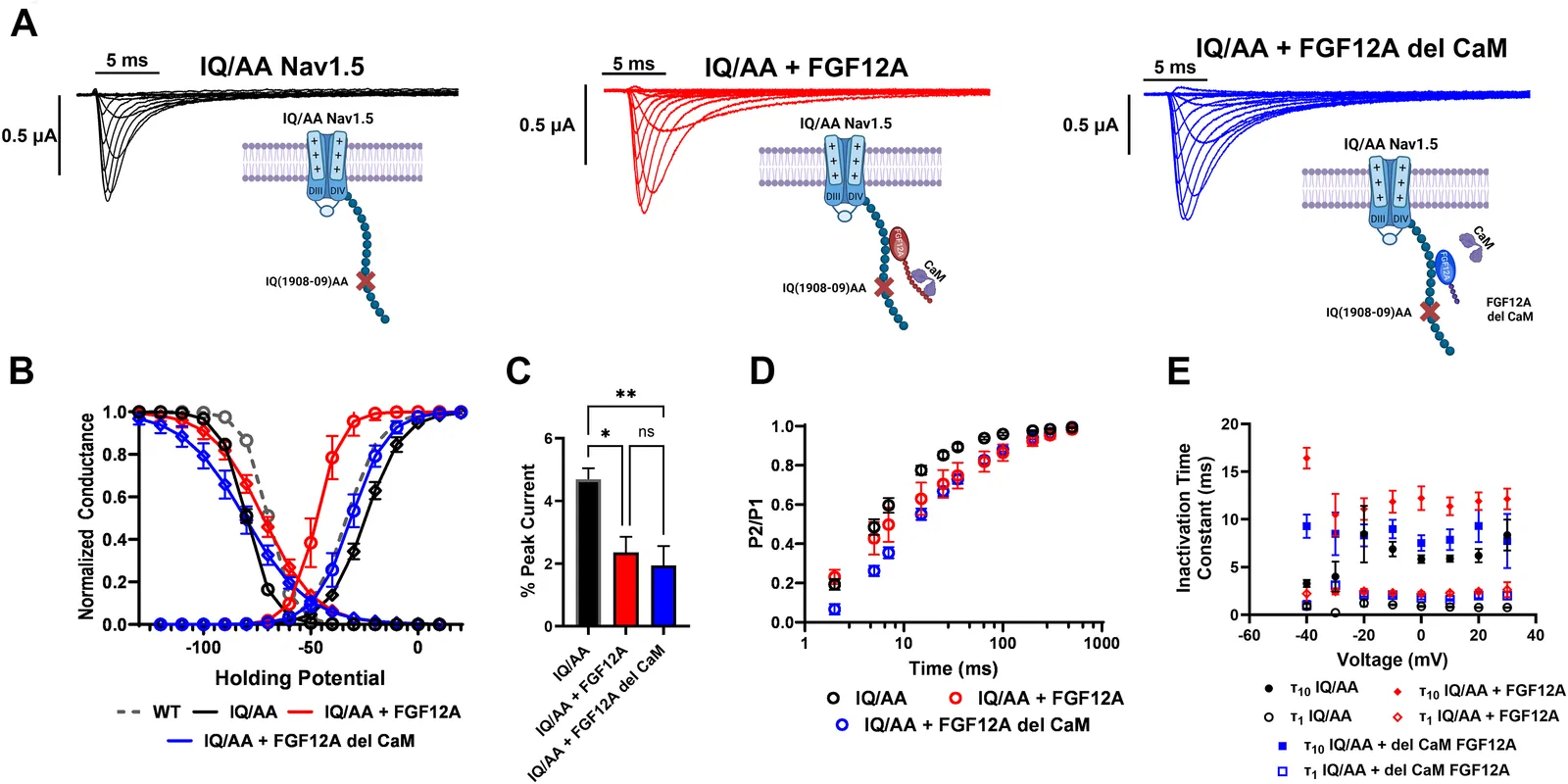
**Removing CaM from the Nav1.5 CTD induces FGF12A-CaM dependent shifts in voltage-dependencies.***A*, representative traces of the IQ(1908-09)/AA Nav1.5 mutation which prevents binding of the CaM protein to the Nav1.5 CTD. Shown with schematic representations of the auxiliary subunits. *B*, mean ± SEM GV and SSI plots with fitted Boltzmann functions. WT Nav1.5 (*dashed gray*), IQ/AA (*black*), IQ/AA + FGF12A (*red*), and IQ/AA + FGF12A del CaM (*blue*). The absence of CaM on the Nav1.5 CTD allows for a more dramatic CaM-dependent shift in activation and inactivation with the presence of WT FGF12A. *C*, persistent late current (I_Na,L_) is significantly lowered regardless of the presence of CaM with FGF12A (Table 1). Percent of I_Na,L_ at −30 mV holding potential analysis is described in Methods. *Error bars* are ±SEM. p-values were calculated with an ordinary one-way ANOVA with Tukey's multiple comparison test (∗*p* = 0.0137, ∗∗*p* = 0.0062). *D*, mean ± SEM recovery from inactivation. *E*, mean ± SEM τ_1_ and τ_10_ inactivation time constants of I_Na_ decay with WT FGF12A showing a significantly higher τ_10_ component. CaM, calmodulin; CTD, C-terminal domain; SSI, steady-state inactivation.

When co-injected with the IQ/AA variant, a difference in SSI voltage-dependence was observed between WT FGF12A and FGF12A del CaM. Only WT FGF12A produced a significant depolarizing shift (+12.3 mV ± 1.79, *p* < 0.0001) compared to the IQ/AA channel (Fig. 2*B* and Table 1). A major hyperpolarizing shift in the GV curve was also observed in WT FGF12A (−15.1 mV ± 2.93, *p* < 0.0001) with FGF12A del CaM shifting the GV V_1/2_ back to WT Nav1.5 values compared to the independent IQ/AA variant (−8.94 mV ± 1.49, *p* < 0.0001) (Fig. 2*B* and Table 1). Similarly, only WT FGF12A significantly increased τ_10_ (+6.49 ms ± 1.11, *p* = 0.0009). However, both WT FGF12A and FGF12A del CaM slightly increased τ_1_ (+2.23 ms ± 0.123, *p* < 0.0001) and (+2.87 ms ± 0.281, *p* = 0.0052) respectively compared to the IQ/AA variant (Fig. 2*E* and Table S1). These results indicate that when CaM is absent from the Nav1.5 CTD, FGF12A produces dramatically larger shifts in inactivation voltage-dependence, consistent with CaM bound to the FGF12A N-terminus driving the effect. The addition of WT FGF12A reduced the I_Na,L_ associated with the IQ/AA variant (−2.33% ± 0.656, *p* = 0.0137) as expected. However, the addition of FGF12A del CaM also decreased I_Na,L_ (−2.75% ± 0.696, *p* = 0.0062) (Fig. 2*C*). Furthermore, there were slight differences in trend of the recovery from inactivation with the addition of either WT FGF12A or FGF12A del CaM compared to the independent IQ/AA variant (Fig. 2*D*). As WT FGF12A and FGF12A del CaM both equally suppressed I_Na,L_, we hypothesized that there are two different mechanisms by which FGF12A interacts with the Nav1.5 channel: CaM-regulated and CaM-independent.

### Altered CaM only impacts WT FGF12A

That FGF12A del CaM and FGF12A scram CaM produced equivalent effects (Fig. 1, *D*–*F*) suggests the observed gating changes are not simply due to the structural consequences of deleting the CaM binding region but does not directly demonstrate that endogenous CaM occupies the WT FGF12A N-terminal site and drives those changes. To assess this directly, we took a complementary approach: rather than modifying FGF12A, we asked whether altering CaM itself would selectively disrupt the gating effects of WT FGF12A. Figure 3*A* illustrates the experimental logic. SSI V_1/2_ should shift when either FGF12A or CaM is altered, but only when FGF12A retains its CaM binding site. FGF12A del CaM, lacking that site, should be insensitive to CaM identity.

**Figure 3:**
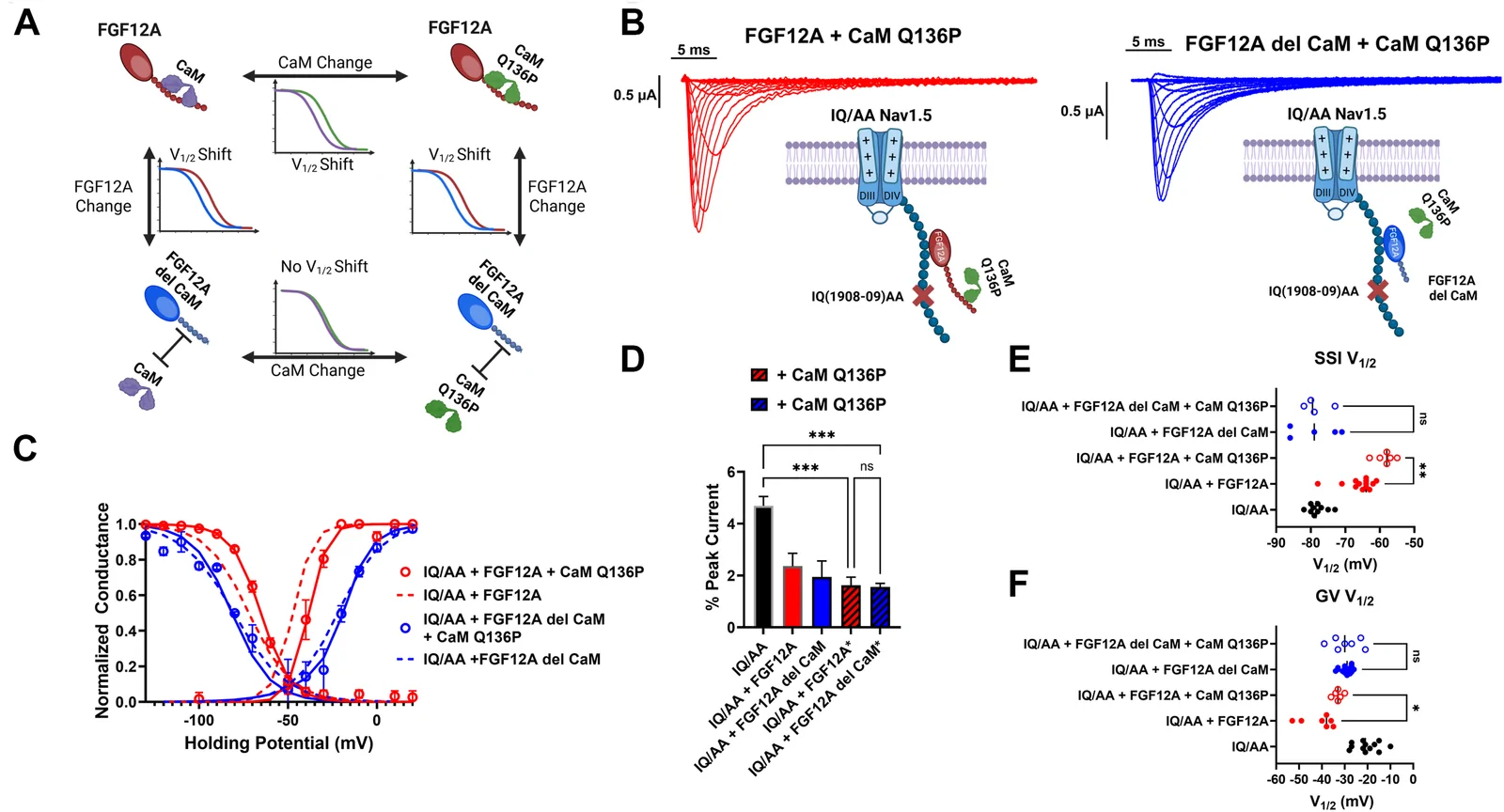
**Altered CaM only impacts WT FGF12A.** All *p*-values were calculated with an ordinary one-way ANOVA with Tukey's multiple comparisons test. *A*, experimental schematic demonstrating the effects of the altered CaM on FGF12A. Changing FGF12A to FGF12A del CaM shifts the SSI V_1/2_. Changing CaM to CaM Q136P shifts the SSI V_1/2_ only with WT FGF12A. Therefore, CaM must interact with FGF12A in order to impact a V_1/2_ shift. *B*, representative traces and schematic views of the IQ(1908-09)/AA variant, with the addition of CaM Q136P and WT FGF12A (*red*) and FGF12A del CaM (*blue*). *C*, mean ± SEM GV and SSI plots with fitted Boltzmann functions. IQ/AA + WT FGF12A + Q136P CaM (*red*), IQ/AA + WT FGF12A + WT CaM (*dashed red*), IQ/AA + FGF12A del CaM + Q136P CaM (*blue*), and IQ/AA + FGF12A del CaM + WT CaM (*dashed blue*) are shown. *D*, percentage of peak current comparing the differences in I_Na,L_ in IQ/AA channels at −30 mV holding potential. *Error bars* are ±SEM. (∗∗∗*p* = 0.001). *E*, *scatter dot plot* with median lines of SSI V_1/2_ (∗∗*p* = 0.0097). *F*, *scatter dot plot* with median lines show GV V_1/2_ (∗*p* = 0.0224). CaM, calmodulin; SSI, steady-state inactivation.

The CaM variant Q136P lies within a CaM EF-hand but is not among the proposed direct contact residues of CaM for FGF12A binding (F19, L32, F92, L105), making it likely to alter but not abolish the FGF12A:CaM interaction (53). The CaM Q136P variant reduces, but does not abolish, Ca^2+^ binding to CaM (55), and it lies outside the CaM–FGF12A contact interface. CaM Q136P is therefore expected to remain bound to the FGF12A N-terminus while altering the Ca^2+^-dependent conformation of the CaM–FGF12A complex, thus modifying rather than eliminating CaM regulation at this site. A comparable effect has been reported for KCNQ1, where CaM Q136P did not disrupt CaM–KCNQ1 binding yet still altered the resulting K^+^ current (37).

CaM Q136P was coinjected with either WT FGF12A or FGF12A del CaM in the IQ/AA Nav1.5 background (Fig. 3*B*). The IQ/AA variant eliminates CaM binding to the Nav1.5 CTD, ensuring that any effect of CaM Q136P on gating must arise through FGF12A rather than the channel itself. Under control conditions, we expect endogenously expressed CaM in the *Xenopus laevis* oocytes to interact with WT FGF12A and WT Nav1.5. We have previously shown that overexpression of CaM variants can impart functional effects on voltage-gated ion channels in *X. laevis* oocytes (37).

As expected with a CaM-regulated mechanism, only WT FGF12A, which has the required CaM-binding site, showed shifts in the V_1/2_ of GV (+2.4 mV ± 0.294, *p* = 0.022) and SSI (+7.35 mV ± 2.07, *p* = 0.0097) with CaM Q136P compared to conditions with endogenous CaM (Fig. 3*C*). No shifts in either the GV (−0.293 mV ± 2.11, *p* > 0.99) or SSI (+0.5 mV ± 2.81, *p* = 0.99) V_1/2_ were observed with FGF12A del CaM and CaM Q136P compared to endogenous CaM (Fig. 3, *E* and *F*). Therefore, as CaM Q136P was prevented from direct interaction with Nav1.5 due to the IQ/AA mutation, the variant’s main route of regulation was through the WT FGF12A’s CaM binding site. We can now observe shifts in SSI V_1/2_ by perturbing either the CaM binding region of FGF12A or CaM itself, but only when the FGF12A binding site is intact.

For the CaM-independent mechanisms, I_Na,L_ was significantly reduced by the presence of both WT FGF12A (−3.14% ± 0.533, *p* = 0.001) and FGF12A del CaM (−3.14% ± 0.556, *p* = 0.001) regardless of which CaM was present/co-injected (Fig. 3*D*).

### Impaired fast inactivation of Nav1.5 illustrates a CaM-independent mechanism of FGF12A

The F1486Q (isoleucine-glutamine-methionine [IQM]) mutation was used to inhibit fast inactivation of the Nav1.5 channel by altering the inactivation motif located on the DIII-DIV linker (56). The IQM variant has a marked difference in fast inactivation as shown in Figure 4*A*. Additionally, the IQM variant increases I_Na,L_ compared to WT Nav1.5 channels (+12.6% ± 1.93, *p* < 0.0001) (Fig. 4*B* and Table 1). The addition of either WT FGF12A (−6.86% ± 2.16, *p* = 0.016) or FGF12A del CaM (−6.14% ± 2.14, *p* = 0.040) measured at −30 mV was sufficient to reduce I_Na,L_ without changes to the peak current expression (Fig. 4*D* and Table 1). This trend was consistent at multiple voltages as shown in Figure 4*D*. Both WT FGF12A and FGF12A del CaM altered the time course of recovery from inactivation of the IQM variant channel in an analogous manner (Fig. 4*C*). GV and SSI curves in addition to inactivation time constants for the IQM variant with and without FGF12A are shown in Supporting Information (Figs. S1 and S3).

**Figure 4:**
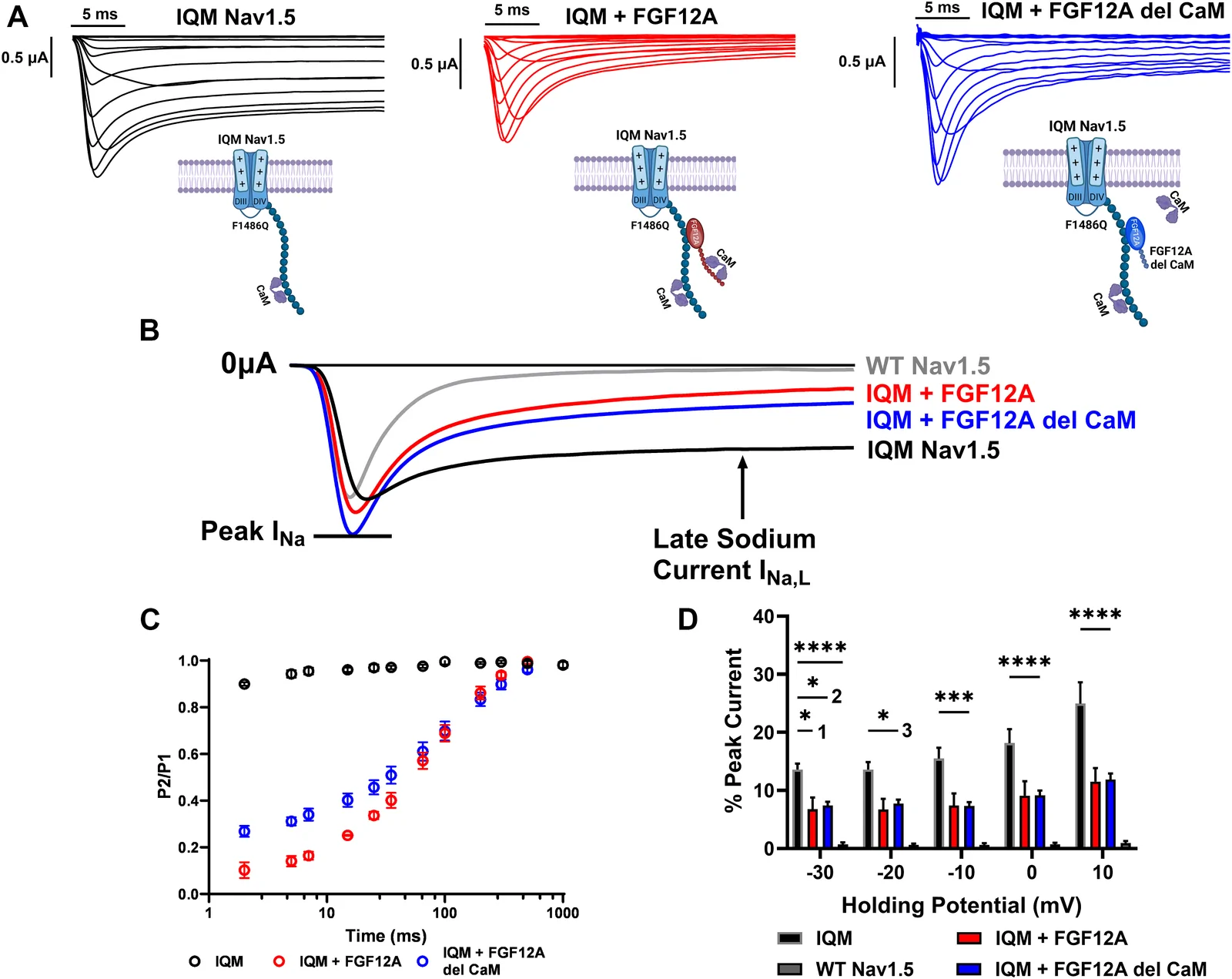
**Inhibiting fast inactivation *via* the IQM mutation demonstrates the CaM-independent mechanism by which FGF12A inhibits I_Na,L_.***A*, representative traces and schematic views of the F1486Q (IQM) variant, with and without auxiliary subunits, that inhibits fast inactivation through altering the DIII-DIV linker responsible for fast inactivation after VSD DIV activation. *B*, comparative traces of Nav1.5 at a step potential of −40 mV. Comparison of late current between Nav1.5 variants includes determining the percentage of the Peak I_Na_ that the I_Na,L_ is. *C*, recovery from inactivation shows that FGF12A with and without CaM significantly alters the relationship. *D*, percentage of peak current comparing the differences in I_Na,L_ in WT and IQM channels at multiple holding potentials (mV). *Error bars* are ±SEM (∗1 *p* = 0.016, ∗2 *p* = 0.0396, ∗3 *p* = 0.0234; ∗∗∗*p* = 0.0004; ∗∗∗∗*p* < 0.0001). All *p*-values were calculated with a Welch ANOVA with Tukey's multiple comparisons test. CaM, calmodulin.

The reduction of I_Na,L_ in the IQM variant by both WT FGF12A and FGF12A del CaM is consistent with the results from the IQ/AA variant (Fig. 2*C*). However, with the IQM variant, CaM is present in the Nav1.5 CTD compared to the IQ/AA variant. Therefore, we conclude that the process by which FGF12A reduces pathogenic I_Na,L_ is CaM-independent as the presence or absence of CaM on either FGF12A or Nav1.5 does not affect FGF12A inhibition of I_Na,L_.

### Nav1.5 long QT syndrome Type 3 variants show a consistent split in CaM mechanisms

We next examined FGF12A regulation of Nav1.5 voltage dependence using LQT3 mutations located on the CTD (Y1795C and S1904L) (57, 58) (Fig. 5*A*). Independently, Y1795C shifts the SSI V_1/2_ leftward compared to the WT Nav1.5 channel (−9.7 mV ± 1.63, *p* < 0.0001) while S1904L shifts the GV V_1/2_ rightward (+11.6 mV ± 2.56, *p* = 0.0041) (Table 1). A depolarizing (right) shift of the V_1/2_ of activation can lead to channel activation at more depolarizing membrane potentials, while a hyperpolarizing (left) shift of inactivation increases the number of channels activated at more depolarized potentials. Both effects can potentially lead to a prolonged cardiac action potential and subsequent arrhythmias (18).

**Figure 5:**
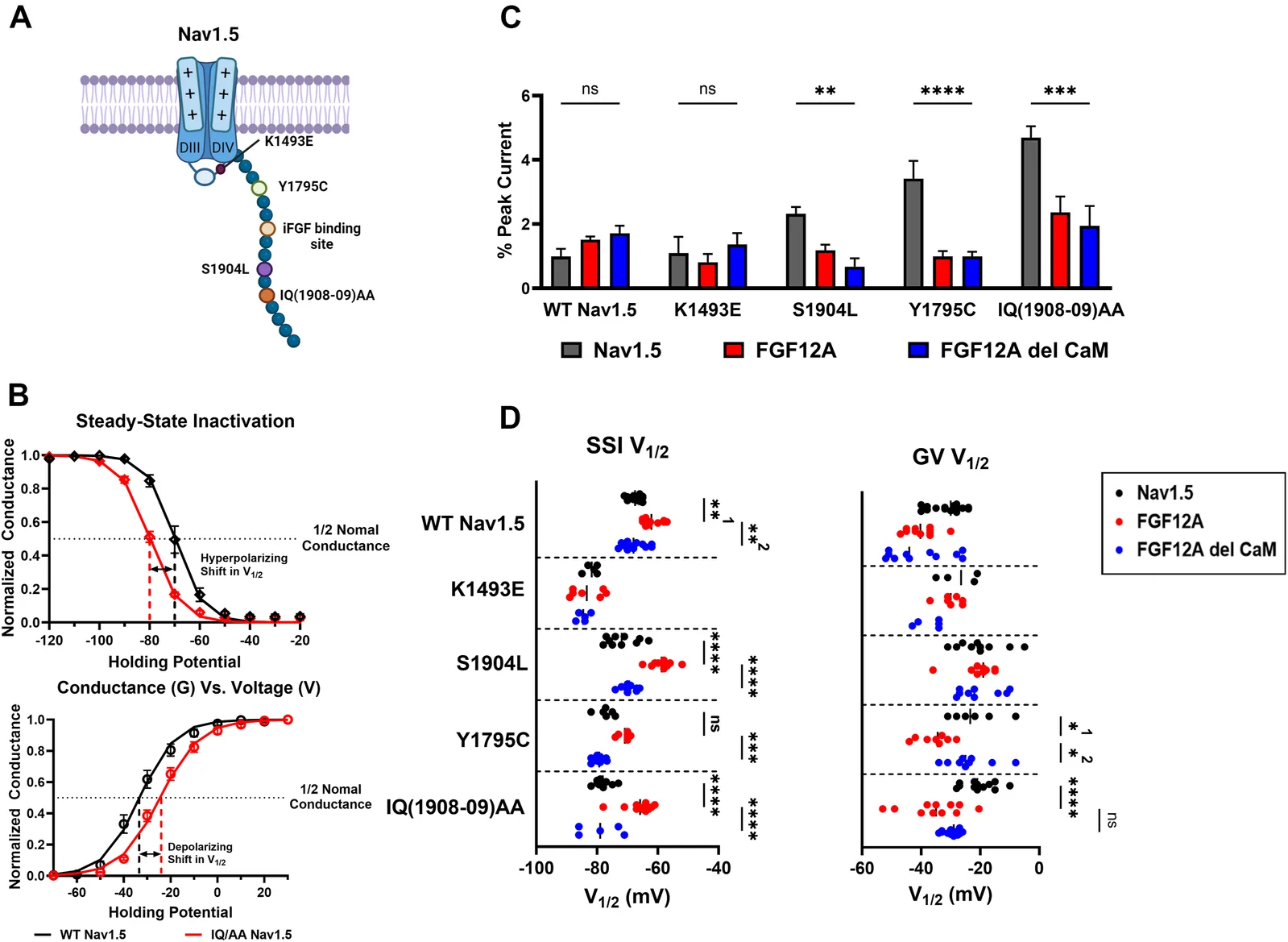
**Altering Nav1.5’s gating kineticsshows both CaM-independent and CaM-dependenteffects.** All *p*-values were calculated with an ordinary one-way ANOVA with Tukey’s multiple comparisons test. *A*, schematic representation of various Nav1.5 mutation locations tested. *B*, example graphs of steady-state inactivation (*top*) and conductance *versus* voltage (*bottom*) that illustrate how the IQ/AA variant (*red*) shifts the V_1/2_ value in either a hyperpolarizing or depolarizing manner compared to WT Nav1.5 (*black*). *C*, FGF12A does not require CaM to decrease the I_Na,L_ from an altered Nav1.5 channel. Each Nav1.5 variant is separated into three groups: no FGF12A (*gray*), with WT FGF12A (*red*), with FGF12A del CaM (*blue*). Bar graph with mean ± SEM shown (∗∗*p* = 0.0058, ∗∗∗*p* = 0.0002, ∗∗∗∗*p* < 0.0001). Data from Figure 2*C* is duplicated here. *D*, FGF12A requires CaM to depolarize the SSI V_1/2_ except when the DIII-DIV linker is unable to interact with the CTD as in the case of K1493E. Additionally, a slight hyperpolarizing shift occurs with GV V_1/2_ only with WT FGF12A. Graph is a *scatter dot plot* with the median line shown. SSI (∗∗1 *p* = 0.0032, ∗∗2 *p* = 0.0019, ∗∗∗*p* = 0.0003, ∗∗∗∗*p* < 0.0001), GV (∗1 *p* = 0.0289, ∗2 *p* = 0.0251, ∗∗∗∗*p* < 0.0001) (Table 1). CTD, C-terminal domain; SSI, steady-state inactivation.

We observed a depolarizing shift in the SSI V_1/2_ with the addition of WT FGF12A to WT Nav1.5 and LQT3 variants while the addition of FGF12A del CaM had no significant shifts in V_1/2_ (Fig. 5*D* and Table 1). We also observed that WT FGF12A induces a hyperpolarizing shift in the GV V_1/2_ of Y1795C, but not in S1904L (Fig. 5*D* and Table 1). Thus, FGF12A alters the two variants in an opposite manner, depolarizing the SSI in S1904L and hyperpolarizing the GV in Y1795C. Additionally, the observation of WT FGF12A affecting SSI or GV while FGF12A del CaM does not is recapitulated with the IQ/AA variant (Fig. 2*B*), which indicates that the mechanism is dependent on CaM being associated with FGF12A.

Conversely, this depolarizing SSI V_1/2_ trend was not shown in the K1493E variant which inhibits the interaction between the Nav1.5 CTD and the DIII-DIV linker (59). The K1493E variant individually shifts the SSI V_1/2_ to hyperpolarized potentials (−14.3 mV ± 1.8, *p* < 0.0001) compared to WT Nav1.5 (Table 1). The addition of either WT FGF12A or FGF12A del CaM does not alter the already shifted SSI curve (Fig. 5*D*). The K1493E variant disrupts the interaction between the Nav1.5 CTD and the DIII-DIV linker (59), and the loss of FGF12A CaM-regulated effects in this background indicates that an intact CTD:linker interface is required for CaM-bound FGF12A to shift inactivation voltage-dependence. This places the CaM-regulated mechanism upstream of, and dependent on, normal CTD-mediated inactivation gating.

We continued to examine the FGF12A effect on the Nav1.5 I_Na,L_. S1904L and Y1795C both increased I_Na,L_ compared to WT Nav1.5 (S1904L: +1.33% ± 0.52, *p* = 0.0085) and (Y1795C: +2.43% ± 0.562, *p* = 0.0038) (Table 1). As predicted, the addition of either WT FGF12A or FGF12A del CaM was sufficient to reduce the pathogenic I_Na,L_ (Fig. 5*C* and Table 1) consistent with our findings with the IQ/AA variant (Fig. 2*C*) and the IQM variant (Fig. 4). Representative traces and other measurements (GV, SSI, recovery from inactivation, and inactivation time constants) for the Nav1.5 variants are shown in the Supporting Information Figures (Figs. S1–S4). Overall, there was a consistent difference between the ability of FGF12A to alter the voltage dependence of inactivation *versus* its suppression of pathogenic I_Na,L_, with the former requiring CaM present on the FGF12A N-terminus.

### FGF12A alters the activation of VSD-IV

To determine the mechanistic basis of the CaM-regulated shift in inactivation, we used voltage-clamp fluorometry (VCF) to measure the voltage-dependence of activation of each of the four VSDs individually with and without FGF12A or FGF12A del CaM. Auxiliary subunits such as FGF13VY and β1 have been shown to shift the voltage-dependence of activation of VSD-I and VSD-IV respectively (27, 31, 47). We therefore performed VCF recordings on Nav1.5 channels tagged at each VSD with co-injection of WT FGF12A or FGF12A del CaM (see Experimental procedures) (60).

Figure 6 shows all four voltage-sensing domains with fluorescence-voltage curves and representative traces. For VSDs one through three (I-III), there were no significant differences between independent WT Nav1.5 (*black*), Nav1.5 with WT FGF12A (*red*), and Nav1.5 with FGF12A del CaM (*blue*) (Fig. 6). With VSD-IV, there was a significant hyperpolarizing shift in the V_1/2_ of VSD-IV activation with WT FGF12A compared to an independent WT Nav1.5 (−21.1 mV ± 1.536, *p* = 0.0052) (Table 2). There was no significant difference between WT Nav1.5 and FGF12A del CaM on the VSD-IV activation, though there is a slight nonsignificant hyperpolarizing trend with the addition of FGF12A del CaM (−9.21 mV ± 3.87, *p* = 0.088) (Table 2). This trend may reflect the contribution of the FGF12 core domain, shared with FGF12B, which has been shown to produce a smaller hyperpolarizing shift (∼10 mV) in VSD-IV activation (47). Therefore, we concluded that the CaM-regulated mechanism of WT FGF12A modulates the voltage-dependence and kinetics of inactivation through only VSD-IV.

**Figure 6:**
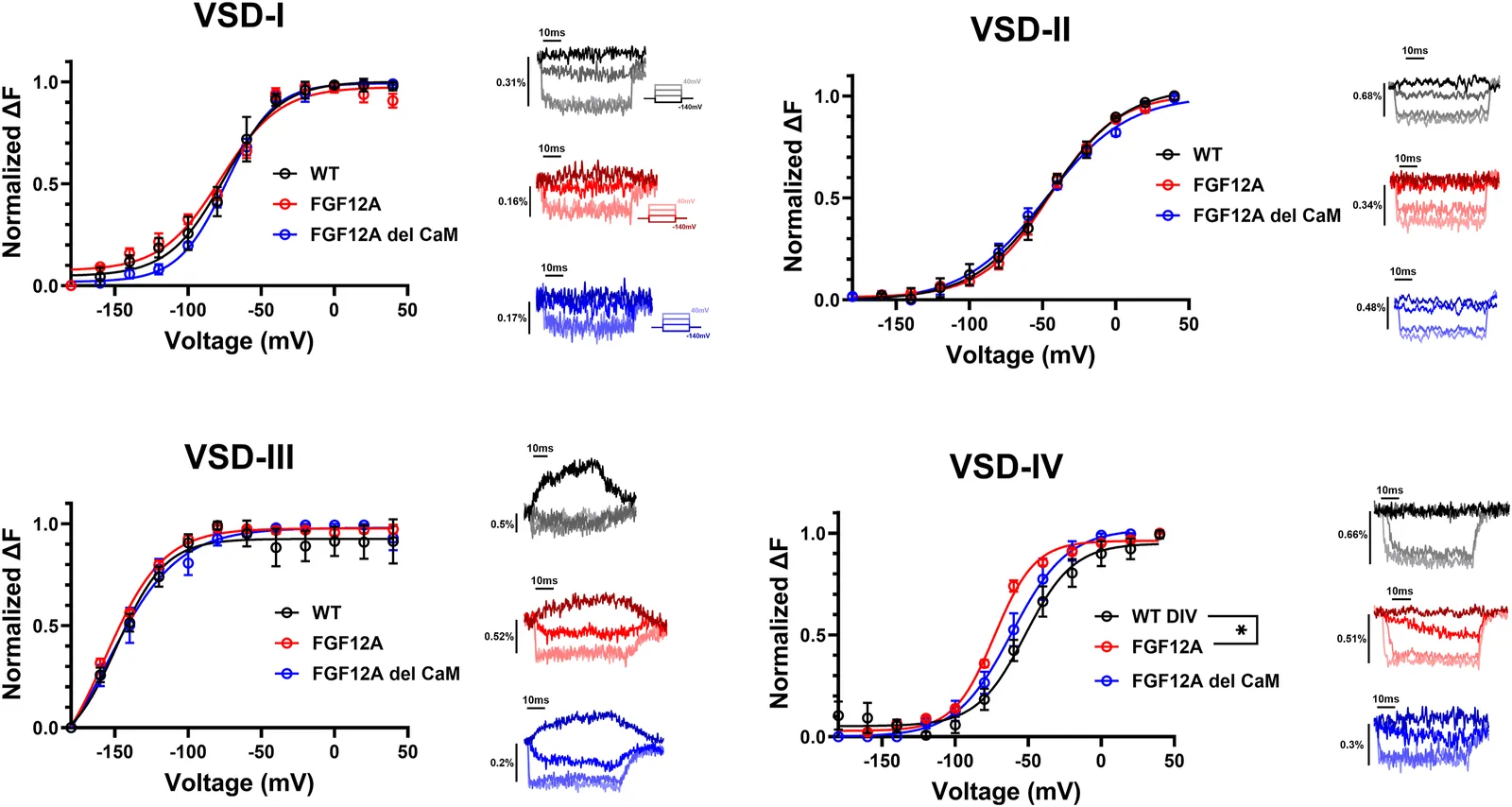
**FGF12A alters the voltage-sensor of domain IV's activation.** Each graph contains the fluorescence-voltage relationship from voltage-clamp fluorometry recordings as described in the Experimental procedures. Each graph contains the mean ± SEM normalized, steady-state fluorescence signal with fitted Boltzmann function, for each voltage-sensor in the four homologous repeats of Nav1.5. Representative fluorescence signals were recorded from *Xenopus laevis* oocytes injected with WT Nav1.5 alone (*black*) or coinjected with WT FGF12A (*red*) or FGF12A del CaM (*blue*). Only traces from the voltage-steps of −140, −80, −20, and 40 mV are shown for clarity. The only significant difference observed was FGF12A hyperpolarizing shift in VSD-IV activation (*bottom right*), compared to WT Nav1.5 (∗*p* = 0.0052). *p*-values were calculated with an ordinary one-way ANOVA with Tukey's multiple comparisons. CaM, calmodulin.

**Table 2:** Voltage-clamp fluorometry recording results for each VSD of Nav1.5 with FGF12A or FGF12A del CaM

Construct	WT Nav1.5	Nav1.5 + FGF12A	Nav1.5 + FGF12A del CaM
VSD-I (n)	5	8	7
V_1/2, VSD-I_ (mV)	−75.40	−77.71	−73.81
±SEM	3.997	6.412	1.790
VSD-II (n)	8	4	5
V_1/2, VSD-II_ (mV)	−44.83	−45.17	−47.85
±SEM	4.085	3.060	4.745
VSD-III (n)	5	5	6
V_1/2, VSD-III_ (mV)	−148.7	−157.0	−156.4
±SEM	9.275	6.409	17.28
VSD-IV (n)	9	8	7
V_1/2, VSD-IV_ (mV)	−52.05	−73.12	−61.26
±SEM	3.875	2.341	1.377

VSD, voltage-sensing domain.

## Discussion

The results presented here reveal that a single auxiliary protein, FGF12A, regulates Nav1.5 through two mechanistically distinct pathways that diverge at the level of CaM occupancy: voltage-dependent inactivation requires CaM bound to the FGF12A N-terminus, while suppression of pathogenic late current does not. The CaM-regulated mechanism operates through the CaM binding site on the FGF12A N-terminus to modulate VSD-IV and alter steady-state inactivation. Nav1.5 gating changes are more pronounced when CaM is also removed from the Nav1.5 CTD *via* the IQ/AA mutation and additionally when the channel is altered through LQT3 mutations. When fast inactivation of the channel is impaired *via* the IQM mutation, FGF12A, regardless of CaM’s presence (WT FGF12A and FGF12A del CaM), can enhance the channel’s inactivation and reduce the pathogenic I_Na,L_. Mechanistically, WT FGF12A shifts the activation of VSD-IV to confer changes to Nav1.5’s inactivation whereas FGF12A del CaM does not. All combined, the results confirm the existence of two distinct mechanisms of action by FGF12A on the Nav1.5 channel.

### FGF12A CaM-independent mechanism reduces pathogenic I_Na,L_

Previous work suggests that FGF12A regulates I_Na,L_ *via* an N-terminal effect on inactivation (45, 46, 48, 54). Chakouri *et al.* 2022 proposed that the FGF12A N-terminal residues of 1 to 30 were independently sufficient to provide a protective effect against pathogenic I_Na,L_ including late current induced by the IQ/AA variant of Nav1.5 (48). Additionally, in Mahling *et al.* 2021, iFGF A-splice variants were proposed to have a long-term inactivation particle on their N-terminus (53). We hypothesize that FGF12A interacts with the channel’s pore *via* its N-terminus (*residues 1–30*) to stabilize fast and slow inactivation. FGF12A can accomplish the same effects with the CaM-binding region completely removed, negating the region’s necessity in reducing I_Na,L_ (Figs. 2 and 5). FGF12B, which is the splice variant of FGF12 without the N-terminal section of FGF12A, does not have the same reduction of I_Na,L_ as FGF12A (48). Goldfarb 2024 described a similar mechanism of canonical FGF12A interaction with the initial N-terminal portion of the protein interacting with the Na^+^ channel’s pore to stabilize the inactivated state of the Nav1.5 channel (45). Our results are consistent with these previous publications as we show FGF12A reduces I_Na,L_ and preserves fast inactivation of the channel (Fig. 4).

### FGF12A CaM-regulated mechanisms alter inactivation kinetics of Nav1.5

Angsutararux *et al.* 2023 found that both the iFGF core region and the N-terminus of the protein are necessary to confer the shifts in inactivation and activation voltage-dependence (47). With regards to FGF12A, we showed that the presence of the CaM binding region altered the FGF12A effect on both the steady-state inactivation and inactivation decay rate. FGF12B, which does not contain a CaM binding region on its N-terminus, can also shift both the SSI and GV in a depolarizing manner (47). FGF12A modestly alters Nav1.5 gating even in the absence of CaM, as seen in Figures 2 and 5. However, FGF12A does require CaM for the full expected shifts in SSI and an increase in the slow time component of inactivation. We found in Figure 6 that FGF12A does significantly alter VSD-IV activation *via* a hyperpolarizing shift. A similar shift is also seen in FGF12B (47), although it is reported to have a less dramatic shift in activation (∼−10 mV shift compared to ∼ −20 mV). This smaller shift is consistent with our finding that FGF12A del CaM produced a nonsignificant hyperpolarizing trend in VSD-IV activation. This result supports the interpretation that the FGF12 core domain can shift VSD-IV activation independently, with CaM occupancy of the N-terminus amplifying the effect. By altering CaM *via* the Q136P mutation, we saw additional evidence of the interaction between FGF12A’s N-terminus and CaM (Fig. 3), as shifts in GV and SSI were observed only with the CaM-bound WT FGF12A.

### Physiological relevance

FGF12A has been shown to reduce pathogenic I_Na,L_ and shift the voltage-dependence of steady-state inactivation in a depolarizing manner. Both effects, through either the CaM-independent or CaM-regulated mechanism, can reduce the duration of the cardiac action potential (18, 61, 62, 63). These effects are most pronounced in the context of pathogenic Nav1.5 variants or loss of auxiliary subunit interactions (18, 26). The CaM-independent suppression of I_Na,L_ positions FGF12A as a constitutive brake on late current, active regardless of Ca^2+^ signaling state. The CaM-regulated inactivation shift, by contrast, provides a Ca^2+^-sensitive layer of control, one that we have shown is dynamically regulated by intracellular Ca^2+^ in a separate study using FRET-based approaches in mammalian cells (64). The conservation of the CaM binding region across all vertebrate iFGF A-splice variants, combined with the finding that endogenous iFGFs compensate for CaM loss at the Nav1.5 CTD in IQ/AA transgenic mice (54), suggests this site evolved as a fail-safe: a CaM reservoir on FGF12A that can substitute for CTD-bound CaM to stabilize fast inactivation when the primary binding site is compromised. The iFGF genes themselves are mostly conserved in all vertebrates with the CaM binding region being located on the N-terminus of all A-splice variants (65, 66). Therefore, the conserved CaM binding region on the A-splice variants of iFGFs is potentially a secondary measure to ensure CaM is present on the Nav1.5 CTD to stabilize fast inactivation.

All A-splice variants of iFGFs have a CaM binding region located on the N-terminus and multiple iFGFs interact with various Nav channels (53). Therefore, the mechanisms shown here could be interpreted as a template for other iFGF:Nav:CaM complexes. For example, the Nav1.2 channel, primarily expressed in the brain, has been shown to interact with the FGF12A isoform, which is also expressed in the nervous system, specifically hippocampal neurons (50, 52, 53). Huang *et al.* 2024 determined that the FGF12A V52H mutation in mice altered the neuronal action potentials of both inhibitory and excitatory neurons, exhibiting how FGF12A can modulate both the maturation of neurons and ion channels (51). Notably, the FGF12A V52H mutation is located within the CaM binding domain, implicating a role of the CaM-regulated FGF12A mechanism. Furthermore, FGF14A differentially modulates multiple Nav channels including Nav1.5 and Nav1.1 and studies have also shown that FGF14 colocalizes to native Nav channels of hippocampal neurons (67). Given the multiple forms of the FGF:Nav complex in different excitable cells, similar CaM-dependent regulatory mechanisms likely operate across other iFGF:Nav complexes, through the interaction of A-type isoforms with Na^+^ channels.

### FGF12A regulation of Nav1.5 gating

Our results show that there are two distinct mechanisms by which FGF12A affects Nav1.5 conduction: a CaM-regulated and a CaM-independent mechanism. Figure 7 represents these two mechanisms in a state-dependent manner. At resting membrane potentials, the Nav1.5 channel is in the closed state: all of its VSDs are in their closed positions, and no Na^+^ ions are flowing (5, 9). Once the membrane is depolarized, the channel moves into the open state: VSD-I to III activate which translocate the coupled S5-S6 helices to open the pore, allowing for Na^+^ ions to flow inward (5, 9). Subsequently, the channel goes into the inactivated state: VSD-IV activates revealing an allosteric pocket, allowing the IFM motif along the DIII-DIV linker to bind (10, 60, 68). The binding drives the S5-S6 helices to constrict, causing fast inactivation of the channel, preventing the flow of Na^+^ ions (12, 14). As detailed above, the fast inactivation of the channel is modulated by the CTD *via* the DIII-DIV linker (14, 15, 16). The CaM-bound FGF12A protein alters the voltage-dependence of steady-state inactivation and the fast inactivation decay rate (Figs. 1 and 4). Specifically, WT FGF12A alters the activation of VSD-IV through the DIII-DIV linker (Fig. 6). For Nav1.5’s fast inactivation, the IQ-bound CaM on the Nav1.5 CTD is required for normal function, but it is not necessary for the CaM-regulated mechanism FGF12A imposes as shown in Figure 2. The FGF12A-bound CaM may function in a similar manner to the IQ-bound CaM in that it stabilizes the Nav1.5 CTD’s interaction with the DIII-DIV linker (59). After fast inactivation, the Nav1.5 CTD dynamically disengages from the DIII-DIV linker (14), freeing the channel pore for occlusion by the FGF12A N-terminal long-term inactivation particle. This stably inactivated state has been hypothesized in previous studies where the N-terminus of iFGF A-splice variants can block open Na^+^ channels to form a stable long-term inactivation state which reduces I_Na,L_ (45, 46). As shown in Figures 2 and 3, CaM is not required on either the Nav1.5 CTD or the FGF12A N-terminus for preservation of fast inactivation or inhibition of pathogenic I_Na,L_.

**Figure 7:**
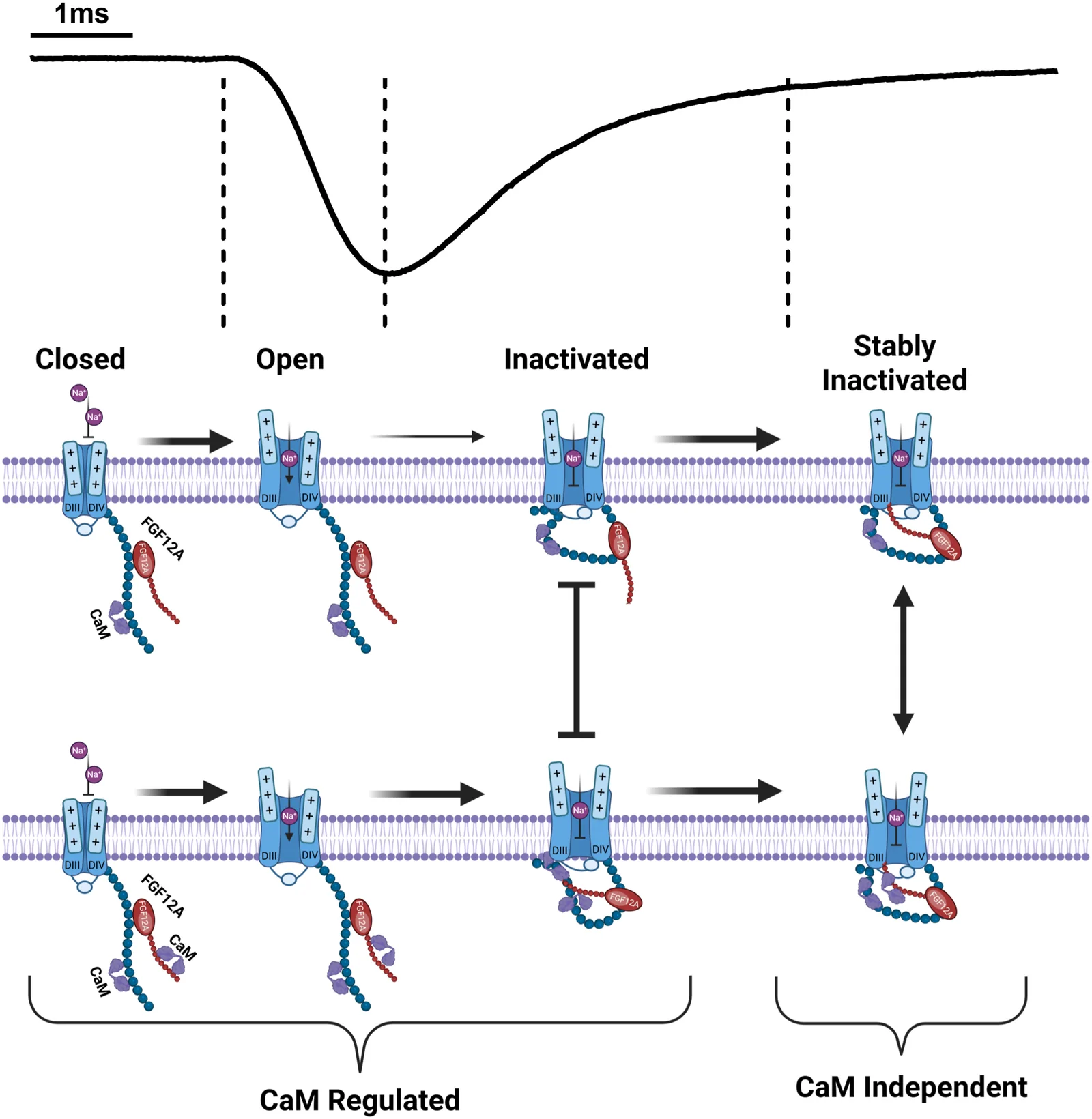
**There are two mechanisms by which FGF12A interacts with the Nav1.5 channel *via* the CTD.** The following states follow the current trace (*top*) and the corresponding time course. Two versions of the states are shown: either with (*bottom*) or without *(middle)* the FGF12A-bound CaM. The IQ-bound CaM is located on all versions of the Nav1.5 states. It is required for normal Nav1.5 function but is not involved in either mechanism. Closed state: All VSDs are in their resting position; no Na+ ions are flowing inward. Open state: VSDs I-III are activated; Na+ ions are flowing inward. Inactivated State: The DIV VSD activates allowing the IFM motif to bind for fast inactivation to the allosteric pocket, thus shifting the coupled S5-S6 helices to close the pore; the Nav1.5 CTD swings to interact with the DIII-DIV linker to modulate VSD-IV activation, which is enhanced *via* the CaM present on the FGF12A N-terminus. CaM is required to be bound to FGF12A in order for FGF12A to modulate the Nav1.5’s inactivated state. Therefore, the two versions of the inactivated state with and without the FGF12A-bound CaM are not considered equivalent. Stably Inactivated State: FGF12A’s N-terminus blocks the pore to stabilize the inactivated state and reduce I_Na,L_. CaM is not required to be present on either the Nav1.5 CTD or FGF12A’s N-terminus for FGF12A’s effect to occur and therefore is a CaM-independent mechanism. The stably inactivated states with and without FGF12A-bound CaM are considered equivalent in the CaM-independent mechanism. CaM, calmodulin; CTD, C-terminal domain.

### Limitations and Ca^2+^ handling

An assumption of these experiments is that the CaM present in *X. laevis* oocytes is in its Ca^2+^-saturated state. Mahling *et al.* (2021) reported that Ca^2+^-saturated CaM binds the N-terminus of A-type iFGFs with high affinity (Kd ∼ 107 nM for FGF12A) (53). Because this interaction is graded rather than all-or-none, full Ca^2+^ saturation strongly favors binding but is not an absolute requirement. During the cardiomyocyte action potential, near-membrane intracellular Ca^2+^ transiently reaches ∼ 2 μM, sufficient to promote CaM-FGF12A interaction (53, 69, 70). Therefore, to avoid introducing additional variables, we altered FGF12A to prevent CaM binding rather than altering the intracellular Ca^2+^ levels in oocytes. We did not directly measure free cytosolic Ca^2+^ under cut-open conditions. However, *X. laevis* oocytes have a reported cytosolic Ca^2+^ concentration of 0.1 to 1 μM with an average of ∼ 100 nM (71, 72), comparable to resting human cardiomyocytes and neurons (69, 73, 74, 75, 76). Furthermore, oocytes have a cytosolic CaM concentration of ∼ 34 μM *versus* ∼ 6 μM in excitable mammalian cells (70, 77). At ∼ 10% activation of CaM at 100 nM Ca^2+^ (53, 78), there would be ∼ 3.4 μM Ca^2+^-CaM available. Even at 1% activation as a conservative lower bound, ∼ 340 nM of available Ca^2+^-CaM exceeds the FGF12A binding K_d_ (107 nM). As total cytosolic CaM is distributed across many binding partners (79), this calculation should be regarded as an upper bound rather than a precise estimate. Empirically, recordings with and without 2 mM EGTA in the internal solution showed no significant differences in GV or SSI curves for either WT FGF12A or FGF12A del CaM (Fig. S5), supporting the interpretation that the FGF12A effect on Nav1.5 gating is preserved across the achievable range of intracellular Ca^2+^, regardless of whether EGTA dialyzes. Functionally, Figure 3 shows that overexpressed CaM Q136P altered Nav1.5 gating when co-injected with WT FGF12A but produced no detectable change with FGF12A del CaM, supporting endogenous CaM engagement at the FGF12A N-terminal site. A Ca^2+^ titration in oocytes comparing gating at minimal and saturating intracellular Ca^2+^ would further refine the Ca^2+^ dependence of the CaM-regulated pathway. The Ca^2+^-dependence of CaM loading onto the FGF12A:Nav1.5 complex is examined directly in our recent HEK293 study (64). However, the central finding of two distinct regulatory mechanisms associated with FGF12A is independent of the precise free Ca^2+^ concentration.

The CaM Q136P overexpression paradigm in *Xenopus* oocytes has been used previously to resolve CaM site occupancy on cardiac ion channels under similar endogenous-CaM conditions. Kang *et al.* 2023 showed that overexpressed CaM Q136P produced detectable changes in I_Ks_ activation kinetics and KCNQ1 membrane trafficking against a background of endogenous WT CaM, demonstrating that CaM Q136P can occupy CaM regulatory sites and exert functional effects despite WT CaM competition (37). The Nav1.5 IQ/AA background used here further reduces competition for Q136P by removing the Nav1.5 C-terminal IQ site, so that overexpressed Q136P need only compete for the FGF12A N-terminal site. We cannot fully exclude a residual contribution from endogenous WT CaM to the gating effects observed, and a definitive test would require a CaM-null or knockdown background that is not available in oocytes. The internal FGF12A del CaM control supports the interpretation that the effect reported here reflects CaM occupancy at the FGF12A N-terminal site, as the gating shift is abolished when this site is deleted. Additional CaM interaction sites on Nav1.5 have been described beyond the C-terminal IQ motif, including sites within the DIII-DIV linker and elsewhere (40, 80, 81). The IQ/AA mutation used here disrupts the primary C-terminal CaM binding site but does not exclude CaM engagement at these alternative sites. Residual CaM occupancy at these sites may contribute to the gating shifts attributed here to the FGF12A N-terminal site, and this represents a limitation of the mutagenesis-based approach. A comprehensive perturbation of all Nav1.5 CaM binding sites was beyond the scope of the present study.

An alternative hypothesis is that with the FGF12A del CaM deletion of the CaM binding region, there are substantial changes in the WT FGF12A structure that would have downstream effects on the Nav1.5 channel itself. This structural change in WT FGF12A compared to FGF12A del CaM could be responsible for the changes seen in Nav1.5 gating function; however, it does not fully explain the split differences between the two groups. As explained in Figure 7 there are two distinct mechanisms associated with FGF12A. The CaM-independent mechanism shows that FGF12A, regardless of CaM’s presence, can inhibit pathogenic late current and rescue fast inactivation (Figs. 2 and 4). Because FGF12A del CaM retains its ability to inhibit I_Na,L_, a function that requires the long-term inactivation particle (*residues 1–30*) (45, 48) but not the N-terminal CaM-binding region, we can conclude that the deletion of residues 38 to 66 did not globally disrupt protein folding. With regards to the CaM-regulated mechanism, as shown in Figure 1, we evaluated a scrambled region version of the FGF12A CaM binding region with similar structural properties (Fig. 1*A*). The scrambled FGF12A showed no significant differences compared to FGF12A del CaM (Table 1). Therefore, the main functional difference between WT FGF12A, FGF12A del CaM, and FGF12A scram CaM is not due to the changes in protein structure, either due to shortening or structural changes in the N-terminus but instead is due to the lack of CaM associated with FGF12A.

Finally, it is possible that the results presented in this study are due solely to changes in the FGF12A protein and not the presence of CaM on the N-terminus. Nonetheless, there is still a split in mechanisms associated with iFGF A-splice variants and the Nav1.5 channel. We have confirmed that the more canonical role of FGF12A in reducing pathogenic I_Na,L_ does not require the CaM-binding region of the FGF12A protein, nor do changes in the length of the N-terminus affect FGF12A’s ability. FGF12A, however, does require the CaM-binding region to substantially alter the voltage-dependencies of activation and inactivation *via* VSD-IV. The V52H mutation, which lies within the CaM-binding region, has been implicated in increased seizure susceptibility in mice (51). Therefore, the CaM-binding region of FGF12A could have additional effects outside of altering Nav1.5 inactivation.

## Experimental procedures

### *Xenopus laevis* oocyte collection

*X. laevis* females with length > 9 cm were purchased from *Xenopus* One Corp. Oocytes were collected from anesthetized frogs in accordance with previously reported protocols (60). All animal handling protocols were approved by Washington University’s Institutional Animal Care and Use Committee (IACUC) and were developed in compliance with the National Institutes of Health (NIH) Guide for the Care and Use of Laboratory Animals.

### Molecular biology

The molecular biology protocols used were adapted from Angsutararux *et al.* 2023 (47). Plasmids encoding human *SCN5A*, FGF12A, and *CALM1* were produced on the pMAX vector (RRID:Addgene_71897). All Nav1.5 point mutations (Table 1), Nav1.5 VCF recording variants (Table 2), FGF12A del CaM (Fig. 1), and CaM Q136P (Fig. 3) were generated using High-Fidelity PCR *via* the Herculase II Fusion DNA Polymerase (Agilent). The scrambled FGF12A (FGF12A Scram CaM) sequence was created with GenScript (Fig. 1). Previously, CaM was hypothesized to be in contact with the amino acids F49 and C55 in FGF12A (53). The scrambled CaM-binding site of FGF12A altered the sequence to remove any phenylalanine and cysteine residues and to conserve the overall structure of the section (Fig. 1*B*). A predictive structure of the WT FGF12A CaM-binding site and the scrambled version are shown in Figure 1*A*. The peptide structure was computed using PEP-FOLD3 (82, 83) and was visualized with PyMOL 2 (RRID:SCR_000305). The scrambled FGF12A plasmid was constructed and packaged by VectorBuilder with the vector ID of VB231023-1405afd. cDNA plasmids were amplified from glycerol *E. coli* stocks with NucleoSpin Plasmid DNA purification (Macherey-Nagel). The cDNA plasmids were linearized with the NucleoSpin Gel and PCR Clean-up kit (Macherey-Nagel). cRNA was then synthesized with the mMESSAGE mMACHINE T7 Transcription kit (Life Technologies) and reconstituted in nuclease-free H_2_O at a concentration of approximately 1 μg/μl.

### Cut-open oocyte voltage-clamp

Collected *X. laevis* oocytes were digested with 24 mg collagenase and 3 mg trypsin inhibitor in 35 ml OR2 buffer (in mM: 82.5 NaCl, 2.5 KCl, 1 MgCl_2_, 5 HEPES, pH 7.6) for 20 min. Oocytes were selected and kept at 5 °C overnight in ND93 solution (in mM: 93 NaCl, 5 KCl, 1.8 CaCl_2_, 1 MgCl_2_, 5 Hepes, 2.5 Na pyruvate, and 1% penicillin/streptomycin, pH 7.4). Oocytes were injected with approximately 50 ng of cRNA with a ratio of 2:1:1 (Nav1.5, β_1_, FGF12A) or 3:1 (Nav1.5:β_1_) for co-expression and incubated for 24 to 48 h at 18 °C prior to recordings. The auxiliary subunit of β_1_ (*SCN1B*) was present in each group for consistency and increased channel expression (28).

A rig and protocol described previously were used for oocyte voltage-clamp recordings (47, 60, 84). Oocytes were kept at 18 °C with an HCC-100A temperature controller (Dagan Corporation) throughout recordings. A CA-1B cut-open amplifier (Dagan Corporation) with corresponding electrodes and bath-guard was connected with a digitizer (1440 Digidata; Molecular Devices) to the computer software Clampex (Molecular Devices; https://www.moleculardevices.com/products/axon-patch-clamp-system/acquisition-and-analysis-software/pclamp-software-suite). The oocytes were recorded in an external solution (in mM, 25 N-methyl-D-glucamine (NMDG), 90 2-(N-Morpholino)ethanesulfonic acid (MES) sodium salt (Na-MES), 20 Hepes, 2 Ca-MES_2_, pH 7.4) and an internal solution (in mM, 105 NMDG, 10 Na-MES, 20 Hepes, 2 EGTA, pH 7.4). To ensure consistent levels of intracellular calcium, EGTA was included in the internal recording solution. The impact of EGTA on endogenous CaM and Na^+^ channel recordings was evaluated and found to be negligible (Fig. S5). AgCl pellets used for electrodes were separated from the recording bath by agar-coated glass bridges, as oocytes have calcium triggered chloride channels (85).

For steady-state activation, oocytes were clamped from −120 to 60 mV for 100 ms with a holding potential of −120 mV. The peak currents were measured, normalized, and divided by the driving force to determine the conductance (G) at each voltage step. The G-V curves were fitted with the Boltzmann Equation (Equation 1):

(1) $$G\left(V\right)=1/\left\{1+\exp\left[-\frac{V-V_{\frac{1}{2}}}{k}\right]\right\}$$

with V_1/2_ defined as the voltage at half-activation and k as the slope.

Ionic currents were measured after P/-8 leak subtraction and membrane capacitance compensation. Cut-open oocyte recordings typically have low leak currents (∼<150–200 nA) as the cut-open guard set-up protects against high non-specific leak currents (31, 84, 86). Our peak current amplitudes were 1 to 3 μA. Cells with imperfect seals or broken membranes were discarded from analysis due to potentially higher leak currents. The percentage of peak current was measured by extracting the peak amplitude at −30 mV and comparing it to the average late Na^+^ current. The late Na^+^ current (I_Na,L_) was the average steady-state current of the protocol’s last 10 ms of the voltage-step protocol at −30 mV.

To measure the inactivation time constants, a two-exponential equation (Equation 2) was fitted to the decay phase of activation at each voltage step:

(2) $$I\left(t\right)=C-A_{1}\;\exp\left(\frac{t}{\tau_{1}}\right)-A_{10}\;\exp\left(\frac{t}{\tau_{10}}\right)$$

where A_1_ and A_10_ are the amplitudes of inactivation and τ_1_ and τ_10_ are the time components on the time scales of 1 ms or 10 ms respectively.

Steady-state inactivation was observed by pre-pulsing the cells from −150 to 20 mV for 200 ms and then stepping to −20 mV to determine the channel availability. Recovery from inactivation was measured with a two-pulse protocol in which the first pulse stepped up from the holding potential of −120 mV to −20 mV for 20 ms. The second pulse was applied after a varying amount of time (2–1000 ms). The ratio between the first peak (P1) and the second (P2) was used to determine channel recovery.

### VCF recordings

Oocytes were prepared in an analogous manner to the cut-open oocyte voltage-clamp protocol previously mentioned. We have previously determined the Nav1.5 point mutations to individually tag each VSD for fluorescence recordings (VSD-I: V215C, VSD-II: S805C, VSD-III: M1296C, VSD-IV: S1618C) (60). After injection, oocytes were incubated in ND93 solution at 18 °C for ∼3 to 6 days before recordings. VCF recordings were conducted in a similar manner as in Angsutararux *et al.* 2023 on the rig previously described above and in Rudokas *et al.* 2014 (47, 84, 87). Cells were placed in a hyperpolarizing solution (in mM: 115 NMDG, 20 Hepes, 2 Ca(OH)_2_, pH to 7.4 with HCl) for 30 s, then in a depolarizing solution (in mM: 0.2 EGTA, 110 KCl, 0.8 CaCl_2_, 1.5 MgCl_2_, 10 Hepes, pH to 7.1 with KOH) for another 30 s to allow access to the cysteine residues for staining. Cells were then labeled for 30 min on ice with 10 μmol/L of methanethiosulfonate-carboxytetramethylrhodamine (Santa Cruz Biotechnology) in depolarizing solution. Recordings of fluorescence signals and ionic currents were simultaneously recorded as previously described (31, 47, 60, 84). Fluorescence traces were baseline corrected for photobleaching and normalized to the steady-state fluorescence trace during voltage-steps. The voltage-dependence of fluorescence change was fitted to the Boltzmann Equation (Equation 1) modified with F(V) substituted into the place of G(V).

### Electrophysiological data analyses

Electrophysiological data and statistical analysis were conducted with Clampfit (Molecular Devices), Prism 10 (GraphPad), and JupyterLab. The statistical significance between experimental groups was determined by a paired *t* test for repeat measurements or an ordinary one-way ANOVA with Tukey’s multiple comparisons test for multi-group comparisons. Cells were excluded from statistical analysis due to small signal-to-noise ratios, fluctuating leak current due to a damaged cell membrane, or an inaccurate recorded membrane potential. Figures and schematics were generated in part in BioRender (RRID:SCR_018361). Blinding was not implemented for data collection or analysis.

## Data availability

All raw experimental data are available upon request. Data are currently located at the Washington University in St Louis Data Repository: https://data.library.wustl.edu/record/108287.

## Supporting information

This article contains supporting information.

## Declaration of generative AI and AI-assisted technologies in the writing process

A large language model (Anthropic Claude, Opus 4.8) was used during preparation of this manuscript to improve writing style. The tool was not used to generate or analyze data, and all AI-assisted text was reviewed and edited by the authors, who take full responsibility for the content of the manuscript.

## Conflict of interest

The authors declare that they have no conflicts of interest with the contents of this article.

## Figures and Tables

**Figure S1:**
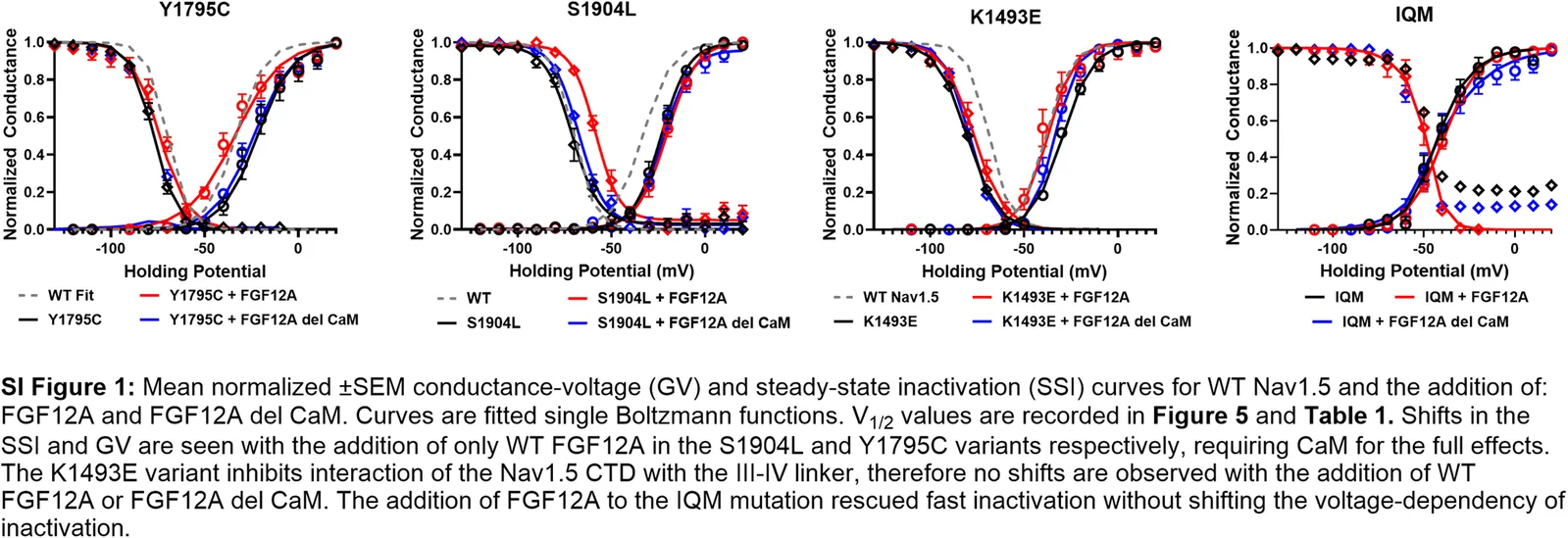


**Figure S2:**
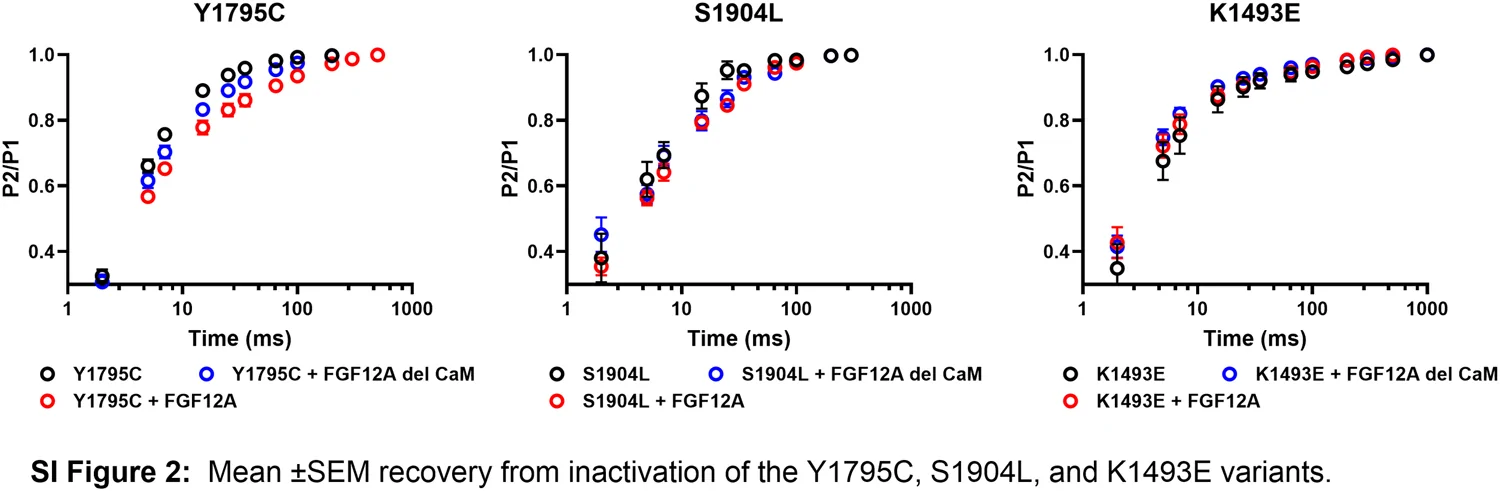


**Figure S3:**
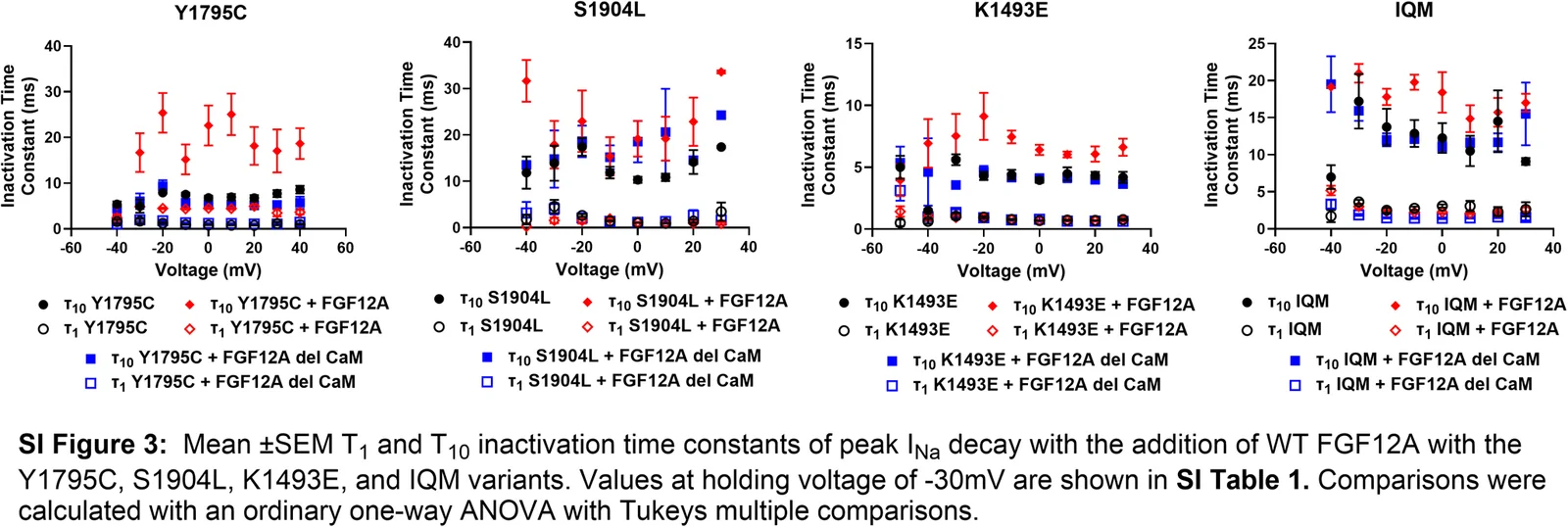


**Figure S4:**
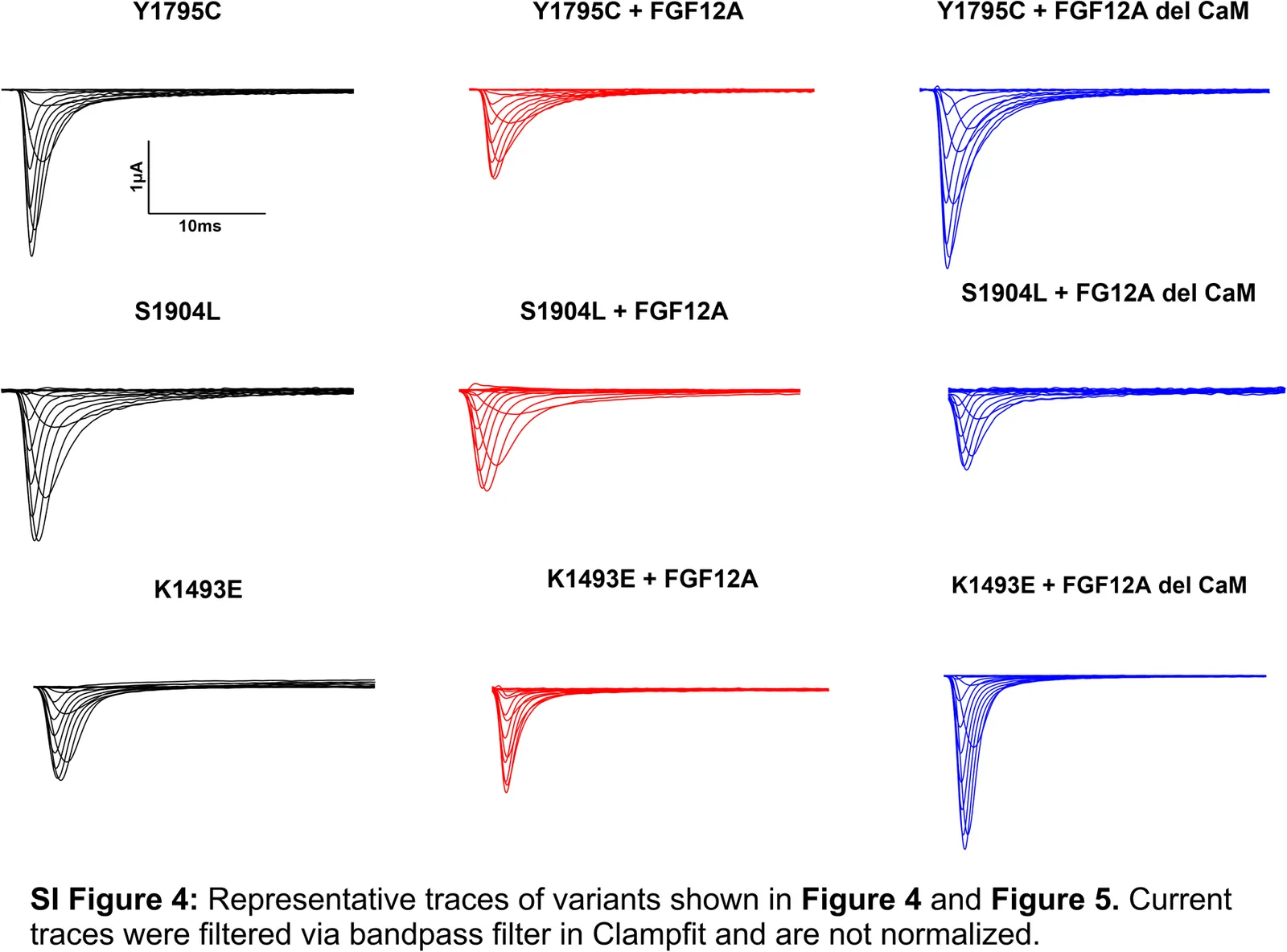


**Figure S5:**
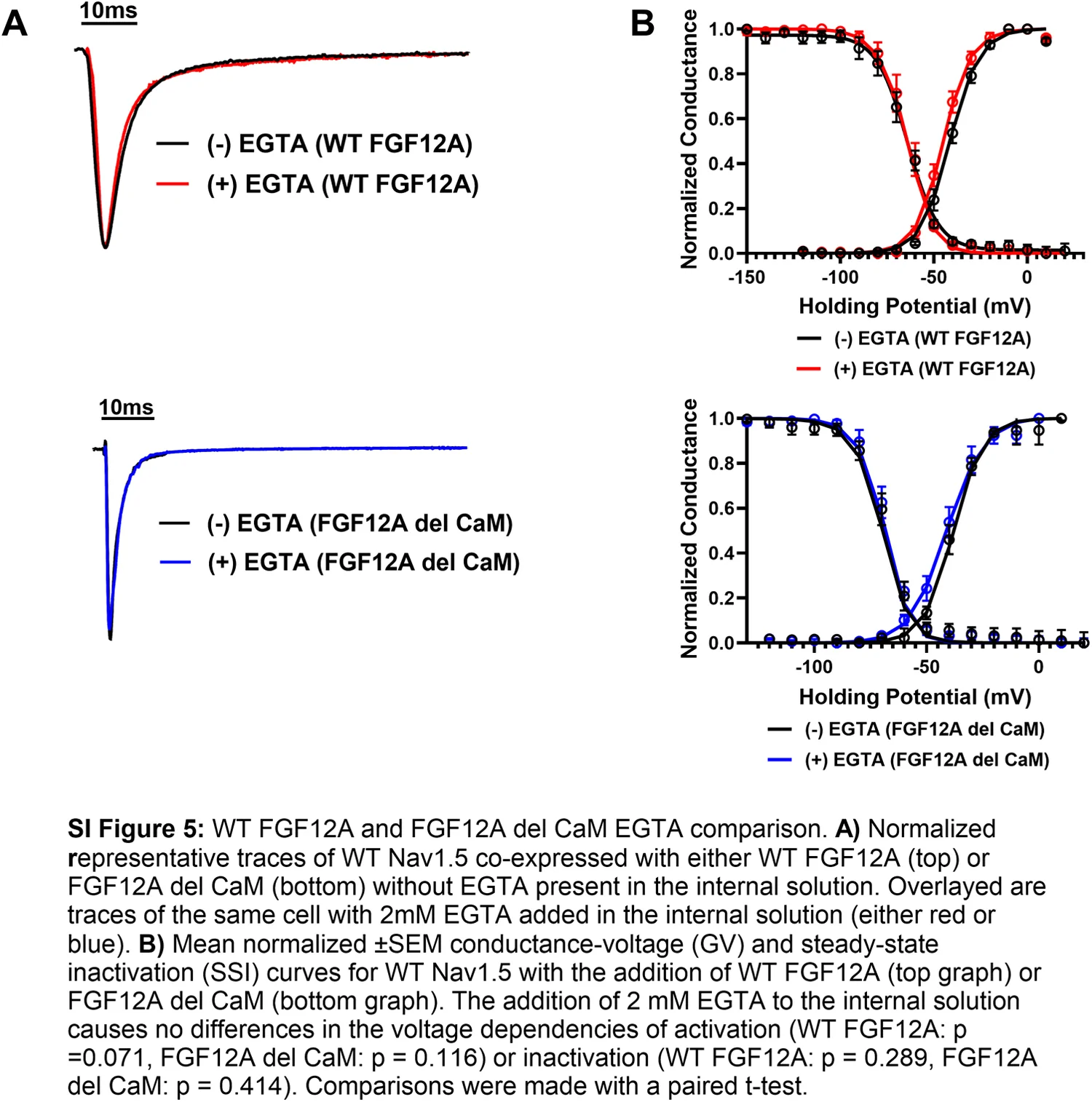


## References

[1] Catterall W.A., Goldin A.L., Waxman S.G. (2005). International union of pharmacology. XLVII. Nomenclature and structure-function relationships of voltage-gated sodium channels. Pharmacol. Rev..

[2] Eijkelkamp N., Linley J.E., Baker M.D., Minett M.S., Cregg R., Werdehausen R. (2012). Neurological perspectives on voltage-gated sodium channels. Brain.

[3] Wang J., Ou S.-W., Zhang Z.-Y., Qiu B., Wang Y.-J. (2018). Molecular expression of multiple Nav1.5 splice variants in the frontal lobe of the human brain. Int. J. Mol. Med..

[4] Catterall W.A. (2023). Voltage gated sodium and calcium channels: discovery, structure, function, and pharmacology. Channels (Austin).

[5] Jiang D., Banh R., Gamal El-Din T.M., Tonggu L., Lenaeus M.J., Pomès R. (2021). Open-state structure and pore gating mechanism of the cardiac sodium channel. Cell.

[6] Yang N., George A.L., Horn R. (1996). Molecular basis of charge movement in voltage-gated sodium channels. Neuron.

[7] Ahern C.A., Payandeh J., Bosmans F., Chanda B. (2016). The hitchhiker’s guide to the voltage-gated sodium channel galaxy. J. Gen. Physiol..

[8] Mangold K.E., Brumback B.D., Angsutararux P., Voelker T.L., Zhu W., Kang P.W. (2017). Mechanisms and models of cardiac sodium channel inactivation. Channels Austin Tex..

[9] Chanda B., Bezanilla F. (2002). Tracking voltage-dependent conformational changes in skeletal muscle sodium channel during activation. J. Gen. Physiol..

[10] Capes D.L., Goldschen-Ohm M.P., Arcisio-Miranda M., Bezanilla F., Chanda B. (2013). Domain IV voltage-sensor movement is both sufficient and rate limiting for fast inactivation in sodium channels. J. Gen. Physiol..

[11] Jiang D., Shi H., Tonggu L., Gamal El-Din T.M., Lenaeus M.J., Zhao Y. (2020). Structure of the cardiac sodium channel. Cell.

[12] Li Z., Jin X., Wu T., Zhao X., Wang W., Lei J. (2021). Structure of human Nav1.5 reveals the fast inactivation-related segments as a mutational hotspot for the long QT syndrome. Proc. Natl. Acad. Sci. U. S. A..

[13] Deschênes I., Trottier E., Chahine M. (2001). Implication of the C-terminal region of the alpha-subunit of voltage-gated sodium channels in fast inactivation. J. Membr. Biol..

[14] Biswas R., López-Serrano A.L., Purohit A., Ramirez-Navarro A., Huang H.-L., Grandinetti G. (2025). Structural basis of human Nav1.5 gating mechanisms. Proc. Natl. Acad. Sci. U. S. A..

[15] Lile S.K., Campbell E.M., Gyawu R.F., Smith J.A., Gwaltney S.R., Johnson C.N. (2025). Potential C-terminal domain interactions of the cardiac voltage gated sodium channel. Sci. Rep..

[16] Shen H., Zhou Q., Pan X., Li Z., Wu J., Yan N. (2017). Structure of a eukaryotic voltage-gated sodium channel at near-atomic resolution. Science.

[17] Wang C., Chung B.C., Yan H., Lee S.-Y., Pitt G.S. (2012). Crystal structure of the ternary complex of a NaV C-terminal domain, a fibroblast growth factor homologous factor, and calmodulin. Structure.

[18] Han D., Tan H., Sun C., Li G. (2018). Dysfunctional Nav1.5 channels due to SCN5A mutations. Exp. Biol. Med..

[19] Mizusawa Y., Wilde A.A.M. (2012). Brugada syndrome. Circ. Arrhythm. Electrophysiol..

[20] Parisi P., Oliva A., Coll Vidal M., Partemi S., Campuzano O., Iglesias A. (2013). Coexistence of epilepsy and Brugada syndrome in a family with *SCN5A* mutation. Epilepsy Res..

[21] Aurlien D., Leren T.P., Taubøll E., Gjerstad L. (2009). New SCN5A mutation in a SUDEP victim with idiopathic epilepsy. Seizure.

[22] Miyazaki A., Sakaguchi H., Aiba T., Kumakura A., Matsuoka M., Hayama Y. (2016). Comorbid epilepsy and developmental disorders in congenital long QT syndrome with life-threatening perinatal arrhythmias. JACC Clin. Electrophysiol..

[23] Johnson J.N., Hofman N., Haglund C.M., Cascino G.D., Wilde A.A.M., Ackerman M.J. (2009). Identification of a possible pathogenic link between congenital long QT syndrome and epilepsy. Neurology.

[24] Wang J., Ou S.-W., Wang Y.-J., Zong Z.-H., Lin L., Kameyama M. (2008). New variants of Nav1.5/SCN5A encode Na+ channels in the brain. J. Neurogenet..

[25] Wu L., Nishiyama K., Hollyfield J.G., Wang Q. (2002). Localization of Nav1.5 sodium channel protein in the mouse brain. Neuroreport.

[26] Abriel H. (2010). Cardiac sodium channel Nav1.5 and interacting proteins: physiology and pathophysiology. J. Mol. Cell. Cardiol..

[27] Angsutararux P., Zhu W., Voelker T.L., Silva J.R. (2021). Molecular pathology of sodium channel beta-subunit variants. Front. Pharmacol..

[28] Martinez-Moreno R., Selga E., Riuró H., Carreras D., Parnes M., Srinivasan C. (2020). An SCN1B variant affects both cardiac-type (NaV1.5) and brain-type (NaV1.1) sodium currents and contributes to complex concomitant brain and cardiac disorders. Front. Cell Dev. Biol..

[29] Moreno J.D., Zhu W., Mangold K., Chung W., Silva J.R. (2019). A molecularly detailed NaV1.5 model reveals a new class I antiarrhythmic target. JACC Basic Transl. Sci..

[30] Zhu W., Wang W., Angsutararux P., Mellor R.L., Isom L.L., Nerbonne J.M. (2021). Modulation of the effects of Class-Ib antiarrhythmics on cardiac NaV1.5-encoded channels by accessory NaVβ subunits. JCI Insight.

[31] Zhu W., Voelker T.L., Varga Z., Schubert A.R., Nerbonne J.M., Silva J.R. (2017). Mechanisms of noncovalent β subunit regulation of NaV channel gating. J. Gen. Physiol..

[32] Yu F.H., Westenbroek R.E., Silos-Santiago I., McCormick K.A., Lawson D., Ge P. (2003). Sodium channel β4, a new disulfide-linked auxiliary subunit with similarity to β2. J. Neurosci..

[33] Crotti L., Johnson C.N., Graf E., De Ferrari G.M., Cuneo B.F., Ovadia M. (2013). Calmodulin mutations associated with recurrent cardiac arrest in infants. Circulation.

[34] Houdusse A., Silver M., Cohen C. (1996). A model of Ca(2+)-free calmodulin binding to unconventional myosins reveals how calmodulin acts as a regulatory switch. Structure.

[35] Ben-Johny M., Yue D.T. (2014). Calmodulin regulation (calmodulation) of voltage-gated calcium channels. J. Gen. Physiol..

[36] Herzog R.I., Liu C., Waxman S.G., Cummins T.R. (2003). Calmodulin binds to the C terminus of sodium channels Nav1.4 and Nav1.6 and differentially modulates their functional properties. J. Neurosci..

[37] Kang P.W., Woodbury L., Angsutararux P., Sambare N., Shi J., Marras M. (2023). Arrhythmia-associated calmodulin variants interact with KCNQ1 to confer aberrant membrane trafficking and function. PNAS Nexus.

[38] Gabelli S.B., Boto A., Kuhns V.H., Bianchet M.A., Farinelli F., Aripirala S. (2014). Regulation of the NaV1.5 cytoplasmic domain by calmodulin. Nat. Commun..

[39] Tidow H., Nissen P. (2013). Structural diversity of calmodulin binding to its target sites. FEBS J..

[40] Wang Z., Vermij S.H., Sottas V., Shestak A., Ross-Kaschitza D., Zaklyazminskaya E.V. (2020). Calmodulin binds to the N-terminal domain of the cardiac sodium channel Nav1.5. Channels(Austin).

[41] Kang P.W., Chakouri N., Diaz J., Tomaselli G.F., Yue D.T., Ben-Johny M. (2021). Elementary mechanisms of calmodulin regulation of NaV1.5 producing divergent arrhythmogenic phenotypes. Proc. Natl. Acad. Sci. U. S. A..

[42] Yan H., Wang C., Marx S.O., Pitt G.S. (2017). Calmodulin limits pathogenic Na+ channel persistent current. J. Gen. Physiol..

[43] Pablo J.L., Pitt G.S. (2016). Fibroblast growth factor homologous factors (FHFs): new roles in neuronal health and disease. Neuroscientist.

[44] Goetz R., Dover K., Laezza F., Shtraizent N., Huang X., Tchetchik D. (2009). Crystal structure of a fibroblast growth factor homologous factor (FHF) defines a conserved surface on FHFs for binding and modulation of voltage-gated sodium channels. J. Biol. Chem..

[45] Goldfarb M. (2024). Fibroblast growth factor homologous factors: canonical and non-canonical mechanisms of action. J. Physiol..

[46] Dover K., Solinas S., D’Angelo E., Goldfarb M. (2010). Long-term inactivation particle for voltage-gated sodium channels. J. Physiol..

[47] Angsutararux P., Dutta A.K., Marras M., Abella C., Mellor R.L., Shi J. (2023). Differential regulation of cardiac sodium channels by intracellular fibroblast growth factors. J. Gen. Physiol..

[48] Chakouri N., Rivas S., Roybal D., Yang L., Diaz J., Hsu A.L. (2022). Fibroblast growth factor homologous factors serve as a molecular rheostat in tuning arrhythmogenic cardiac late sodium current. Nat. Cardiovasc. Res..

[49] Mahling R., Hegyi B., Cullen E.R., Cho T.M., Rodriques A.R., Fossier L. (2025). De novo design of a peptide modulator to reverse sodium channel dysfunction linked to cardiac arrhythmias and epilepsy. Cell.

[50] Biadun M., Karelus R., Krowarsch D., Opalinski L., Zakrzewska M. (2024). FGF12: biology and function. Differentiation.

[51] Huang J., Sun C., Zhu Q., Wu G., Cao Y., Shi J. (2024). Phenotyping of FGF12AV52H mutation in mouse implies a complex FGF12 network. Neurobiol. Dis..

[52] Wildburger N.C., Ali S.R., Hsu W.-C.J., Shavkunov A.S., Nenov M.N., Lichti C.F. (2015). Quantitative proteomics reveals protein–protein interactions with fibroblast growth factor 12 as a component of the voltage-gated sodium channel 1.2 (Nav1.2) macromolecular complex in Mammalian brain. Mol. Cell. Proteomics.

[53] Mahling R., Rahlf C.R., Hansen S.C., Hayden M.R., Shea M.A. (2021). Ca2+-saturated calmodulin binds tightly to the N-terminal domain of A-type fibroblast growth factor homologous factors. J. Biol. Chem..

[54] Abrams J., Roybal D., Chakouri N., Katchman A.N., Weinberg R., Yang L. (2020). Fibroblast growth factor homologous factors tune arrhythmogenic late Na_V_1.5 current in calmodulin binding–deficient channels. JCI Insight.

[55] Wang K., Brohus M., Holt C., Overgaard M.T., Wimmer R., Van Petegem F. (2020). Arrhythmia mutations in calmodulin can disrupt cooperativity of Ca2+ binding and cause misfolding. J. Physiol..

[56] West J.W., Patton D.E., Scheuer T., Wang Y., Goldin A.L., Catterall W.A. (1992). A cluster of hydrophobic amino acid residues required for fast Na(+)-channel inactivation. Proc. Natl. Acad. Sci. U. S. A..

[57] Bankston J.R., Sampson K.J., Kateriya S., Glaaser I.W., Malito D.L., Chung W.K. (2007). A novel LQT-3 mutation disrupts an inactivation gate complex with distinct rate-dependent phenotypic consequences. Channels(Austin).

[58] Rivolta I., Abriel H., Tateyama M., Liu H., Memmi M., Vardas P. (2001). Inherited Brugada and long QT-3 syndrome mutations of a single residue of the cardiac sodium channel confer distinct channel and clinical phenotypes. J. Biol. Chem..

[59] Gade A.R., Marx S.O., Pitt G.S. (2020). An interaction between the III-IV linker and CTD in NaV1.5 confers regulation of inactivation by CaM and FHF. J. Gen. Physiol..

[60] Varga Z., Zhu W., Schubert A.R., Pardieck J.L., Krumholz A., Hsu E.J. (2015). Direct measurement of cardiac Na+ channel conformations reveals molecular pathologies of inherited mutations. Circ. Arrhythm. Electrophysiol..

[61] Abriel H. (2007). Roles and regulation of the cardiac sodium channel Nav1.5: recent insights from experimental studies. Cardiovasc. Res..

[62] Moreau A., Krahn A., Gosselin-Badaroudine P., Klein G., Christé G., Vincent Y. (2013). Sodium overload due to a persistent current that attenuates the arrhythmogenic potential of a novel LQT3 mutation. Front. Pharmacol..

[63] Moreau A., Gosselin-Badaroudine P., Boutjdir M., Chahine M. (2015). Mutations in the voltage sensors of domains I and II of Nav1.5 that are associated with arrhythmias and dilated cardiomyopathy generate gating pore currents. Front. Pharmacol..

[64] Woodbury L., Li A., Angsutararux P., Marras M., Wagner E., Silva J.R. (2026). FGF12A confers calcium sensitivity to Nav1.5 inactivation via dynamic calmodulin recruitment. J. Physiol..

[65] Goldfarb M. (2005). Fibroblast growth factor homologous factors: evolution, structure, and function. Cytokine Growth Factor Rev..

[66] Satou Y., Imai K.S., Satoh N. (2002). Fgf genes in the basal chordate Ciona intestinalis. Dev. Genes Evol..

[67] Lou J.-Y., Laezza F., Gerber B.R., Xiao M., Yamada K.A., Hartmann H. (2005). Fibroblast growth factor 14 is an intracellular modulator of voltage-gated sodium channels. J. Physiol..

[68] Gardill B.R., Rivera-Acevedo R.E., Tung C.-C., Okon M., McIntosh L.P., Van Petegem F. (2018). The voltage-gated sodium channel EF-hands form an interaction with the III-IV linker that is disturbed by disease-causing mutations. Sci. Rep..

[69] Sutanto H., Lyon A., Lumens J., Schotten U., Dobrev D., Heijman J. (2020). Cardiomyocyte calcium handling in health and disease: insights from *in vitro* and *in silico* studies. Prog. Biophys. Mol. Biol..

[70] Wu X., Bers D.M. (2007). Free and bound intracellular calmodulin measurements in cardiac myocytes. Cell Calcium.

[71] Busa W.B., Nuccitelli R. (1985). An elevated free cytosolic Ca2+ wave follows fertilization in eggs of the frog, Xenopus laevis. J. Cell Biol..

[72] Oron Y., Dascal N. (1992). Regulation of intracellular calcium activity in *Xenopus* oocytes. Methods Enzymol..

[73] Marks A.R. (2013). Calcium cycling proteins and heart failure: mechanisms and therapeutics. J. Clin. Invest..

[74] Berridge M.J. (1998). Neuronal calcium signaling. Neuron.

[75] Walters G.C., Usachev Y.M. (2023). Mitochondrial calcium cycling in neuronal function and neurodegeneration. Front. Cell Dev. Biol..

[76] Ross W.N. (2012). Understanding calcium waves and sparks in central neurons. Nat. Rev. Neurosci..

[77] Cartaud A., Ozon R., Walsh M.P., Haiech J., Demaille J.G. (1980). Xenopus laevis oocyte calmodulin in the process of meiotic maturation. J. Biol. Chem..

[78] Yoder J.B., Ben-Johny M., Farinelli F., Srinivasan L., Shoemaker S.R., Tomaselli G.F. (2019). Ca2+-dependent regulation of sodium channels NaV1.4 and NaV1.5 is controlled by the post-IQ motif. Nat. Commun..

[79] Black D.J., Tran Q.-K., Persechini A. (2004). Monitoring the total available calmodulin concentration in intact cells over the physiological range in free Ca2+. Cell Calcium.

[80] Sarhan M.F., Tung C.-C., Van Petegem F., Ahern C.A. (2012). Crystallographic basis for calcium regulation of sodium channels. Proc. Natl. Acad. Sci. U. S. A..

[81] Johnson C.N., Potet F., Thompson M.K., Kroncke B.M., Glazer A.M., Voehler M.W. (2018). A mechanism of calmodulin modulation of the human cardiac sodium channel. Structure.

[82] Herrera-Bravo J., Farías J.G., Contreras F.P., Herrera-Belén L., Beltrán J.F. (2022). PEP-PREDNa+: a web server for prediction of highly specific peptides targeting voltage-gated Na+ channels using machine learning techniques. Comput. Biol. Med..

[83] Thévenet P., Shen Y., Maupetit J., Guyon F., Derreumaux P., Tufféry P. (2012). PEP-FOLD: an updated de novo structure prediction server for both linear and disulfide bonded cyclic peptides. Nucleic Acids Res..

[84] Rudokas M.W., Varga Z., Schubert A.R., Asaro A.B., Silva J.R. (2014). The Xenopus oocyte cut-open vaseline gap voltage-clamp technique with fluorometry. Jove J. Vis. Exp..

[85] Weber W.-M. (1999). Endogenous ion channels in oocytes of Xenopus laevis: recent developments. J. Membr. Biol..

[86] Taglialatela M., Toro L., Stefani E. (1992). Novel voltage clamp to record small, fast currents from ion channels expressed in Xenopus oocytes. Biophys. J..

[87] Zhu W., Varga Z., Silva J.R. (2016). Molecular motions that shape the cardiac action potential: insights from voltage clamp fluorometry. Prog. Biophys. Mol. Biol..

