# Supplementary material for: Calmodulin acts as a selective switch in FGF12A-mediated regulation of the cardiac sodium channel Nav1.5
Source: J Biol Chem. 2026 Sep 17;302(11):113565. doi: 10.1016/j.jbc.2026.113565 (PMC13691571; doi:10.1016/j.jbc.2026.113565)
Supplement: Supplementary file 1 [file mmc1.docx]

**Supporting Table 1**: Fitted inactivation time constant values for τ_1_ and τ_10_ at -30mV for all Nav1.5 variants with(out) FGF12A or FGF12A del CaM. Values are means with ±SEM fitted from the double exponential equation 2 (see methods) of the decay phase of I_Na_.

| **Variant** | **Decay inactivation (n)** | **τ_1_** | **±SEM** | **τ_10_** | **±SEM** |
| --- | --- | --- | --- | --- | --- |
| Nav1.5 | 4 | 2.78 | 0.379 | 3.93 | 1.34 |
| Nav1.5 + FGF12A | 5 | 1.50 | 0.167 | 9.92 | 0.751 |
| Nav1.5 + FGF12A del CaM | 4 | 1.20 | 0.145 | 4.43 | 1.07 |
| Nav1.5 + FGF12A Scram CaM | 5 | 1.20 | 0.738 | 5.54 | 1.51 |
| IQ/AA | 5 | 0.228 | 0.0031 | 4.01 | 1.57 |
| IQ/AA + FGF12A | 4 | 2.46 | 0.314 | 10.5 | 2.18 |
| IQ/AA + FGF12A del CaM | 7 | 3.10 | 0.336 | 8.49 | 2.24 |
| IQM | 7 | 3.64 | 0.274 | 17.2 | 3.67 |
| IQM + FGF12A | 7 | 3.01 | 0.243 | 21.0 | 1.25 |
| IQM + FGF12A del CaM | 7 | 1.89 | 0.128 | 15.9 | 1.36 |
| K1493E | 5 | 0.973 | 0.0969 | 5.61 | 0.44 |
| K1493E + FGF12A | 6 | 0.939 | 0.157 | 7.57 | 1.79 |
| K1493E + FGF12A del CaM | 7 | 1.36 | 0.238 | 3.57 | 0.139 |
| S1904L | 8 | 4.54 | 1.45 | 13.9 | 3.78 |
| S1904L + FGF12A | 7 | 1.56 | 0.595 | 16.2 | 5.73 |
| S1904L + FGF12A del CaM | 5 | 4.08 | 1.24 | 13.0 | 4.73 |
| Y1795C | 5 | 1.69 | 0.469 | 4.96 | 1.44 |
| Y1795C + FGF12A | 8 | 4.86 | 0.163 | 16.7 | 4.24 |
| Y1795C + FGF12A del CaM | 8 | 1.20 | 0.353 | 6.04 | 1.73 |
|  |  |  |  |  |  |
